# A Potential Probiotic *Lactobacillus plantarum* JBC5 Improves Longevity and Healthy Aging by Modulating Antioxidative, Innate Immunity and Serotonin-Signaling Pathways in *Caenorhabditis elegans*

**DOI:** 10.3390/antiox11020268

**Published:** 2022-01-28

**Authors:** Arun Kumar, Tulsi Joishy, Santanu Das, Mohan C. Kalita, Ashis K. Mukherjee, Mojibur R. Khan

**Affiliations:** 1 Molecular Biology and Microbial Biotechnology Laboratory, Division of Life Sciences, Institute of Advanced Study in Science and Technology (IASST), Guwahati 781035, Assam, India; arunkumar@iasst.res.in (A.K.); tulsi.joishy@iasst.res.in (T.J.); santanudas@iasst.res.in (S.D.); director@iasst.gov.in (A.K.M.); 2 Department of Biotechnology, Gauhati University, Guwahati 781014, Assam, India; mckalita@gauhati.ac.in; 3 Department of Molecular Biology and Biotechnology, School of Sciences, Tezpur University, Tezpur 784028, Assam, India

**Keywords:** longevity, lactic acid bacteria, aging biomarkers, SKN-1 transcription factor, p38 MAPK signaling, behavior, intestinal integrity, mitochondria

## Abstract

Since the hypothesis of Dr. Elie Metchnikoff on lactobacilli-mediated healthy aging, several microbes have been reported to extend the lifespan with different features of healthy aging. However, a microbe affecting diverse features of healthy aging is of choice for broader acceptance and marketability as a next-generation probiotic. We employed *Caenorhabditis elegans* as a model to understand the potential of *Lactobacillus plantarum* JBC5 (LPJBC5), isolated from fermented food sample on longevity and healthy aging as well as their underlying mechanisms. Firstly, LPJBC5 enhanced the mean lifespan of *C. elegans* by 27.81% compared with control (untreated). LPBC5-induced longevity was accompanied with better aging-associated biomarkers, such as physical functions, fat, and lipofuscin accumulation. Lifespan assay on mutant worms and gene expression studies indicated that LPJBC5-mediated longevity was due to upregulation of the skinhead-1 (*skn-1*) gene activated through p38 MAPK signaling cascade. Secondly, the activated transcription factor SKN-1 upregulated the expression of antioxidative, thermo-tolerant, and anti-pathogenic genes. In support, LPJBC5 conferred resistance against abiotic and biotic stresses such as oxidative, heat, and pathogen. LPJBC5 upregulated the expression of intestinal tight junction protein ZOO-1 and improved gut integrity. Thirdly, LPJBC5 improved the learning and memory of worms trained on LPJBC5 compared with naive worms. The results showed upregulation of genes involved in serotonin signaling (*ser-1*, *mod-1*, and *tph-1*) in LPJBC5-fed worms compared with control, suggesting that serotonin-signaling was essential for LPJBC5-mediated improved cognitive function. Fourthly, LPJBC5 decreased the fat accumulation in worms by reducing the expression of genes encoding key substrates and enzymes of fat metabolism (i.e., *fat-5* and *fat-7*). Lastly, LPJBC5 reduced the production of reactive oxygen species and improved mitochondrial function, thereby reducing apoptosis in worms. The capability of a single bacterium on pro-longevity and the features of healthy aging, including enhancement of gut integrity and cognitive functions, makes it an ideal candidate for promotion as a next-generation probiotic.

## 1. Introduction

The aging population has a profound impact on the global economy [1]. Healthy aging refers to the “process of developing and maintaining the functional ability that enables well-being in older age” has been a recent concern [2]. The functional abilities constitute physiological, cognitive, metabolic, and immunological functions in the later stages of life [3]. Older age is considered an important risk factor for many comorbidities, including cancer, diabetes, obesity, neurodegenerative disorders, cardiovascular diseases, and infections [4]. The aging process is influenced by genetics, medical history, pharmacological and dietary interventions [5,6,7,8]. During the past couple of decades, there has been a significant surge in research on prolongevity and healthy aging. Several studies on dietary interventions in both invertebrates (worms and flies) and vertebrate hosts (mice and humans) have shown to promote healthy aging [8,9,10].

*Caenorhabditis elegans* has become a choice as a model for aging research due to its shorter life cycle (2–3 weeks) and aging-related conserved pathways with humans [11]. The significant pathways such as p38 mitogen-activated protein kinase (p38 MAPK) and DAF-2/DAF-16 pathways regulate longevity, resistance against oxidative stress, and defense against pathogens in worms [12,13,14]. These mechanisms are evolutionarily conserved in models from worms to mice [15]. Many studies have shown that these microbivore worms (i.e., feeds on microbes) can explain how a microbial diet could influence longevity, development, reproduction, fat accumulation, cognition, and imparts resistance against biotic and abiotic stresses [11].

Probiotic microbes are known to improve the host’s health by antimicrobial effect, nutritional supplementation, immunomodulation, and maintaining the healthy microbiota within the host’s gut, thereby also promote healthy aging [16]. A century ago, Dr. Elie Metchnikoff observed that lactobacilli-containing yogurt consumption led to increased lifespan and better health of Bulgarian farmers [17]. It was the first observation on probiotics-induced longevity. *Lactobacillus* spp. are the most important bacteria that colonize the gastrointestinal tract of humans since birth [18]. Studies suggest that the lactobacilli population decreases with age, and supplementation of probiotic lactobacilli to older adults improves their health by modulating gut microbiota composition, enhancing immunity and gut health [19,20,21]. Previous reports indicated the role of lactobacilli on longevity with different features of healthy aging such as resistance to oxidative stress and toxicity, protection against pathogenic infections, reducing fat accumulation, improving cognition, alleviating inflammation, metabolic disorders, and neurodegenerative diseases [12,13,14,22,23,24,25,26,27,28,29,30,31,32]. However, a single microbe affecting these diverse features of healthy ageing will increase the acceptance and marketability as a next generation probiotic.

In our previous research on bacteria of curds prepared using boiled milk and raw milk from dairy farms of Assam, India [33], a potential probiotic bacterium was isolated, and it was taxonomically characterized as *Lactobacillus plantarum* strain JBC5 (LPJBC5). In this work, we have demonstrated that LPJBC5 promotes longevity and diverse aspects of healthy aging, including delayed age-related physical functions, reduced fat accumulation, improved resistance to abiotic and biotic stress, gut integrity, cognition functions, mitochondrial functions and reduced apoptosis in *C. elegans*. Our study indicates that bacterium LPJBC5 affects the diverse features of healthy aging, making it an ideal candidate for promotion as next-generation probiotics.

## 2. Materials and Methods

### 2.1. Materials

All microbiological growth media, including deMan, Rogosa and Sharpe (MRS), Nutrient agar and Luria-Bertini (LB) agar were obtained from Himedia, India. The bacterial control food *E. coli* OP50 (OP50), *C. elegans* Bristol wild-type strain N2 and its mutants, including DR1572 *daf-2* (e1368), AU1 *sek-1* (ag1), KU25 *pmk-1* (km25), GR1307 *daf-16* (mgDf50), AU3 *nsy-1* (ag3), and EU31 *skn-1*(zu135) and EU1 *skn-1* (zu67) were obtained from Caenorhabditis Genetics Center (CGC), University of Minnesota, USA. All experiments were performed with self-fertilizing hermaphrodite strains of *C. elegans*. The pathogenic strain *Staphylococcus aureus* MTCC 3160 was obtained from Microbial Type Culture Collection, CSIR-Institute of Microbial Technology (CSIR-IMTECH), Chandigarh, India. The epithelial intestinal cell line HT-29 was procured from National Center of Cell Sciences, Pune, India. The genomic extraction kit was procured from Sigma, Germany. Total RNA was isolated using RNAeasy mini kit (Invitrogen, Waltham, MA, USA) and reverse transcribed to cDNA using Verso cDNA synthesis Kit (Thermo Scientific, Waltham, MA, USA).

### 2.2. Bacterial Strains and Growth Conditions

LPJBC5 was inoculated and cultured in MRS broth at 30 °C for 18 h, whereas OP50 was cultured in Luria-Bertini broth overnight at 37 °C. The pathogenic strain *S. aureus* was inoculated and cultured in Nutrient broth for overnight at 37 °C. All these bacterial strains were cultured under shaking conditions at 150 rpm.

### 2.3. Identification for the Presence of Probiotic Marker Genes in LPJBC5

The presence of probiotic marker genes encoding species-specific collagen-binding protein and bile salt hydrolase was confirmed in LPJBC5, as previously described [34] (Table S1). Additionally, *L. plantarum* specific sequence and anti-microbial gene (plantaricin-biosynthetic gene) was amplified and sequenced in LPJBC5 (Table S1) [35,36]. The genomic DNA was extracted using a genomic extraction kit, and its concentration was determined using a NanoDrop^TM^ 2000c spectrophotometer (Thermo Scientific, USA). The PCR reaction was set up with a total reaction mixture of 25 μL containing 2.5 μL of 10× Taq buffer, 1.5 U of Taq DNA polymerase (Sigma, Darmstadt, Germany), 1.5 mM of MgCl_2_, 100 μM of dNTP mixture, 10 pmol of each primer pair and 50 ng of bacterial DNA. The PCR was performed using the following conditions in a PCR thermal cycler (Eppendorf, Hamburg, Germany): 96 °C for 5 min, followed by 35 cycles of 94 °C for 30 s, 57 °C for 30 s, 72 °C for 1 min, and 72 °C for 5 min. The amplified product was run on 1.5% agarose gel and imaged under a UV transilluminator (Vilber, Collégien, France). Sequencing of purified PCR amplified fragments was performed with Macrogen Inc. (Seoul, Korea). The DNA sequences were subjected to BLAST analysis and submitted to NCBI (National Center for Biotechnology Information, Bethesda, Rockville, MD, USA) database. 

### 2.4. Phylogenetic Tree

To determine the phylogenetic relationships of strain LPJBC5, the 16S rDNA gene sequence was compared with other strains of *Lactobacillus* and one outgroup genera *Enterococcus faecium* ATCC 19434. The identification of phylogenetic neighbours and calculation of pairwise 16S rDNA gene sequence similarities and aligned using phylogenetic tree analysis software (MEGA 7 version) [37]. Neighbour-joining algorithms were used for the reconstruction of the phylogenetic tree. Bootstrap analysis was conducted to determine the confidence limits of the branching.

### 2.5. Survival to the Gastrointestinal (GIT) Transit

The simulated gastric juices were prepared by using a method described by Conway et al. [38]. Firstly, the grown LPJBC5 culture (10^9^ CFU/mL) was washed twice with PBS and resuspended in GIT juice (0.3% pepsin) at pH 2.0 and incubated at 37 °C for 0 h, 1 h, and 3 h. Secondly, the grown LPJBC5 culture (10^9^ CFU/mL) was washed with PBS, resuspended in intestinal juice (0.1% porcine pancreatin, Sigma, Germany), and further adjusted to pH 8.0. The treated LPJBC5 cells were incubated at 37 °C for 4 h. Thirdly, the acid tolerance of LPJBC5 was evaluated at different pH range from 1.0, 3.0, and 7.0 (considered as control) of PBS. The LPJBC5 culture (10^9^ CFU/mL) was added to 10 mL of different pH solutions and incubated at 37 °C for 4 h. Each experiment was performed in triplicate, and after completion of incubation time, the viable LPJBC5 cells were counted for each treatment by spread-plating on MRS agar plates and incubated at 37 °C for 24–48 h.

### 2.6. Assay for Bile Acid Tolerance

The tolerance to Bile salt of the isolate LPJBC5 was measured using a procedure described by Vinderola and Reinheimer [39]. Briefly, the solutions of bile salts were prepared using Ox-gall bile (Sigma, Darmstadt, Germany) with 0.3% and 1% concentrations, and PBS served as control. 10^9^ CFU/mL cells of grown isolate LPJBC5 were further added to 10 mL bile salt solutions and incubated at 37 °C for 0 h and 4 h. The viable LPJBC5 cells were counted for each treatment by spread-plating on MRS agar plates and incubated at 37 °C for 24–48 h.

### 2.7. Adhesion to Intestinal Cells

The adhesion ability of LPJBC5 to epithelial intestinal cell line HT-29 was tested using a procedure discussed in Ayeni et al. [40]. The adhesion of isolate LPJBC5 was calculated as the number of adhered cells to HT-29 cell line after 4 h of incubation in comparison with the initial population in the DMEM suspension.

### 2.8. Longevity Assay

The Nematode Growth Medium (NGM) plates (60 mm) were seeded with 10^9^ CFU/mL of OP50 or LPJBC5 suspended in M9 buffer (22 mM KH_2_PO_4_, 42 mM Na_2_HPO_4_, and 86 mM NaCl) [41]. The synchronization of worms were performed by a modified procedure described by Stiernagle [42]. “Briefly, the NGM plates with sufficient eggs of self-fertilizing hermaphrodite worms were washed with deionized water (Millipore, Burlington, MA, USA) and pipetted into a 5 mL conical centrifuge tube. The deionized water was adjusted to 3.5 mL in a conical centrifuge tube and 1.5 mL volume of a mixture of 1 mL sodium hypochlorite bleach and 0.5 mL of 5 N NaOH was added. The tubes were then vortexed for approximately 2 min and centrifuged at 7500 rpm to pellet down the released eggs. The supernatant was thrown, and pellet of worms was washed twice with deionized water. At last, the remaining pellet was suspended in approximately 0.1 mL of deionized water and transferred to NGM plate seeded with standard bacterial food *E. coli* OP50”. The longevity assays were performed with young adult wild-type N2 and mutant worms. The longevity experiment was performed in 3 technical replicates in which each NGM plate contained 50 worms and plated were further incubated at 20 °C. The worms were counted every 24 h and transferred to new NGM plates every two days to maintain sufficient bacterial food. The worms were excluded from lifespan analysis if it adheres to the wall of the plate, showing abnormal death due to progeny hatch inside their body and vulva explosion [41]. Survival analysis was conducted using the Kaplan–Meier method in OASIS 2 software, and their mean lifespan was calculated [43].

### 2.9. Determination of Pharynx Pumping and Locomotor Activity

The pharynx pumps and locomotor activity of worms were determined using a protocol described by Nakagawa et al. [12]. The synchronized worms were initially grown on OP50 for 2 days and further cultured throughout adulthood for 14 days on OP50 or LPJBC5. The worms were then transferred to a new OP50-seeded NGM plate 30 min before recording their pumping rates or locomotor ability. The number of pharynx pumps rates were measured for 30 s using a stereo zoom microscope (SMZ1270, Nikon, Tokyo, Japan) [12]. The locomotory rate was measured by counting the body bends per minute on the NGM plate using a stereo zoom microscope [12]. Ten worms were assessed for each bacterial strain, and 3 replicates were used for each bacterium.

### 2.10. Developmental Rate Assay

The developmental rate of worms fed on both bacterial diets was analyzed using a procedure described by Soukas et al. [44]. About 100 age-synchronized worms were allowed to lay eggs on OP50-seeded NGM plate for 30 min and synchronized using a bleaching protocol. Once the worms reached their young adult stage, 20 worms per bacterial strains were individually transferred to OP50 or LPJBC5 seeded NGM plates in triplicates and continuously observed under a stereo zoom microscope (SMZ1270, Nikon, Japan) every 30 min until we observed the first laid egg.

### 2.11. Measurement of Body Size

The body sizes of OP50 and LPJBC5-fed worms were determined as described by Zanni et al. [45]. The age-synchronized worms were initially cultured on OP50 for 3 days and transferred to OP50 or LPJBC5 (10^9^ CFU/mL) seeded plates. The images were captured using a stereo zoom microscope (SMZ1270, Nikon, Japan) every 24 h until the age of day 7, and body size was determined using ImageJ software (National Institutes of Health, MD, USA). Ten worms grown in each bacterial strain were used for measuring the body size, and for assuring reproducibility, the experiment was performed in triplicate.

### 2.12. Colonization Efficiency

The synchronized eggs of worms were transferred to NGM-plates seeded with OP50 or LPJBC5. A total of 10 worms per bacterial strain were washed and further lysed to check their colonization efficiency on the third and eighth days. The whole nematode lysate of LPJBC5 or OP50-fed worms were plated in triplicates on their respective culture media containing plates [46]. The colony-forming units (CFU) were counted for each bacterial strain after incubating at their respective cultural conditions.

### 2.13. Aging Pigment Accumulation

The study on the accumulation of aging pigment or lipofuscin levels of worms was conducted as described by Kwon et al. [41]. Briefly, the worms were cultured on OP50 or LPJBC5 for 14 days, washed, and transferred onto 3% (*w*/*v*) agarose pads on a glass slide for confocal microscopy (TCS SPE, Leica, Wetzlar, Germany). Ten worms were quantified for each bacterial strain using ImageJ software, and three replicates were used for each bacterium.

### 2.14. Brood Size

The brood size of worms was determined as described by Zanni et al. [45]. The number of progenies per nematode was calculated, and the experiment was conducted in triplicate for both bacterial strains.

### 2.15. Determination of Fat Accumulation

Age-synchronized young adult worms were cultured on OP50 or LPJBC5 for 14 days. Further, the accumulation of body fat was examined by Oil Red O (ORO) staining in worms according to a protocol described by Nakagawa et al. [12]. The lipid accumulation was observed and imaged using a compound microscope (10× and 20× objective lens) (AX10, Carl Zeiss, Jena, Germany), and the relative staining intensity was quantitated with ImageJ software. Ten worms were analyzed to quantify the accumulation of fat for each bacterial strain, and three replicates were used for each bacterium.

### 2.16. Food Preference and Learning Memory

The binary choice assay was performed to observe the food preference of worms on different bacterial diets as described by Bendesky et al. [47]. The assay was conducted on 60 mm NGM plate. A total of 25 μL of LPJBC5 or OP50 (10^9^ CFU/mL) was seeded at the opposite sides of the plates and dried for 1 h under laminar airflow. Day 3 worms (30 worms/plate) were individually transferred onto the center of the NGM plate with equidistant lawns of OP50 and LPJBC5 and counted worms in both bacterial lawns after 4 h, and the experiment was conducted in triplicate. The choice index (CI) of bacterial food preference in worms was calculated as follows:

Choice index (CI) = Number of worms in LPJBC5 – Number of worms in OP50/Total number of worms used in an assay

CI = −1.0 shows complete food preference for control food OP50.

CI = +1.0 shows complete food preference for testing bacteria LPJBC5.

CI = 0.0 shows equal distribution of food preference for both OP50 and LPJBC5.

For training, day 3 worms (*n* = 30) were cultured onto an NGM plate seeded with LPJBC5 and cultured for 4 h at 20 °C. The worms were further washed thrice with M9 buffer and transferred onto the center of the plate seeded with equidistant lawns of OP50 and LPJBC5. The memory index was calculated as:

Memory index = CI (Trained LPJBC5) − CI (Naive OP50)

A total of 30 worms per plate were analyzed in each preference assay, and the experiment was performed in triplicates.

### 2.17. Thermotolerance and Oxidative Stress-Resistance Assay

Age-synchronized worms were initially cultured on OP50 until the young adult stage, then individually transferred onto NGM plates seeded with OP50 or LPJBC5 for 3 days at 20 °C. For thermotolerance assay, these cultured plates were maintained at 35 °C, and viable worms were counted every hour until all of them died [48].

For the oxidative stress-resistance assay, day 3 young worms were grown on OP50 or LPJBC5 for three days and individually picked and transferred to an M9 solution containing 100 mM paraquat (Sigma Aldrich, St. Louis, MO, USA) and incubated at 20 °C [49]. The viable worms were counted after 8 h. Fifty worms were transferred to three wells against each treatment, and the experiment was performed in triplicates for each treatment. The lipofuscin level was also measured after 8 h of incubation in 100 mM paraquat using confocal microscopy as described previously (see material and methods 2.13). Ten worms were quantified for each treatment using ImageJ software, and three replicates were used for each treatment.

### 2.18. Determination of Resistance against Pathogenic Bacterial Infections

Age-synchronized young adult worms were initially grown on OP50-seeded plates, and further individually transferred on OP50 or LPJBC5 seeded NGM plates for 3 days. These worms were then individually transferred to NGM plates seeded with the pathogenic bacterium *S. aureus* (10^9^ CFU/mL), and incubated at 20 °C [41]. Survival of worms was recorded every 24 h. A total of 50 worms were used in each plate, and the experiment was performed in triplicate for each bacterium.

### 2.19. Measurement of Intestinal Integrity against Pathogenic Infection (Smurf Assay)

The effect of LPJBC5 against intestinal barrier infections was evaluated according to Kim and Moon [50]. Briefly, the age-synchronized young adult worms grown on OP50 were cultured on OP50 or LPJBC5 for five days. The worms were then individually transferred to NGM plates seeded with pathogen *S. aureus* for 2 days. To observe intestinal permeability in response to pathogenic exposure, OP50 was heat-killed at 70 °C for 2 h (confirmed by plating on LB-agar). The heat-killed OP50 culture was centrifuged, and the pellet was resuspended in blue food dye (5% *w*/*v*) and shaken for 3 h at 35 rpm at room temperature. The worms were then grown in a liquid NGM medium containing heat-killed OP50 stained with blue food dye for 3 h. The worms were washed several times in M9 buffer, anesthetized in the same buffer containing 25 mM levamisole (L-025, Sigma, Germany) and imaged with a compound microscope (AX10, Carl Zeiss, Germany) at 10× and 20× magnifications. The blue food dye in the body of worms (*n* = 10) was quantitatively analyzed using ImageJ software under 10× magnification.

### 2.20. RNA Isolation, cDNA Synthesis, and Quantitative Reverse Transcription-Polymerase Chain Reaction (qRT-PCR)

The age-synchronized worms were grown on OP50 for 2 days at 20 °C. The young adult worms were then transferred to NGM plates seeded with LPJBC5 or OP50 for 24 h. Approximately 500 worms per group were collected and washed to extract the total RNA. About 1 μg of extracted RNA was reverse transcribed to cDNA by using Verso cDNA synthesis Kit. qRT-PCR was performed to measure the expression of the genes using SYBR Green (Applied Biosystems, USA) in a real-time PCR machine (Applied Biosystem, USA). The genes include DAF-2/DAF-16 (*daf-2* and *daf-16*), p38 MAPK (*nsy-1*, *sek-1* and *pmk-1*) and its downstream genes (*skn-1* and *skn-1b*), FOXA transcription factor *pha-4*, phase-2 detoxification genes GSTs (*gst-4*, *gst-7* and *gst-10*), catalases (*ctl-1* and *ctl2*), Thioredoxin-1 (*trx-1*) and SODs (*sod-1*, *sod-2* and *sod-3*), heat-shock proteins HSPs (*hsp-60* and *hsp-70*, *hsp-16.1* and *hsp-16.2*), genes encoding key substrates and enzymes of fat metabolism (*fat-5*, *fat-6* and *fat-7*), serotonin signaling genes (*ser-1*, *mod-1*, and *tph-1*), innate immunity genes [saponin-like proteins (*spp-1* and *spp-7*), lysozymes (*lys-1* and *lys-8*), C-type lectin (CLEC) domain-containing proteins (*clec-60* and *clec-85*) and antibacterial factor (ABF) (*abf-1*, *abf-2* and *abf-3*)], tight junction protein zonula occludin *zoo-1*, mitochondrial DNA (mtDNA) encoded NADH-ubiquinone oxidoreductase chain 1 (*nd-1*), anti-apoptotic (*ced-9*) and pro-apoptotic (*ced-3* and *ced-4*) genes. The primer sequences of the studied genes are listed in Table S2. The experiment was performed with three replicates. The relative expression of each gene was analyzed using 2^−ΔΔCt^ method [51]. The housekeeping gene *act-1* was used to normalize the expression of each gene.

The expression of innate immune genes was studied by pre-culturing the young adult worms on LPJBC5 or OP50 for 24 h, and then transferred to NGM plates seeded with *S. aureus*. After 12 h of post-infection, the worms were collected and washed three times with M9 buffer. RNA isolation, cDNA synthesis, and qRT-PCR were performed as described above.

### 2.21. GSH/GSSG Assay

Glutathione Assay Kit (Promega, Madison, WI, USA) was used to individually measure reduced glutathione (GSH) and oxidized glutathione (GSSG) in day-14 worms grown on OP50 or LPJBC5 as per the manufacturer’s instructions. It is a luminescent based system to detect both GSH and GSSG in worms. In brief, the worms were transferred to the wells of a 96 well flat bottom plate. A 50 μL volume of total or oxidized glutathione lysis reagent was added to each group of well in a 96-well plate, and the plates were shaken at 3000 rpm for 5 min at room temperature. The luciferin generation reagent was added (50 μL/well) and the plates were incubated for 30 min at room temperature. The luciferin detection reagent was added (100 μL/well) to each well and luminescence was recorded in a multimode plate reader (Varioskan Flash, Thermo Scientific, USA) at 562 nm. The total level of luminescence was detected in both LPJBC5-fed and OP50-fed worms and the ratio of GSH to GSSG was calculated. One hundred worms were used for each bacterial-treated group, and the experiment was performed in triplicate.

### 2.22. SOD Activity Assay

SOD Assay Kit-WST (19160, Sigma, USA) was used to measure superoxide dismutase (SOD) activity in day 14 worms grown on OP50 or LPJBC5 as per the manufacturer’s instructions. Additionally, the required preparation of protein extracts and further determination of SOD activity was performed using a procedure described by Nakagawa et al. [12]. One hundred worms per group were used to prepare the protein extract, and the experiment was performed in triplicate.

### 2.23. Measurement of Intracellular ROS Generation

The fluorescent non-polar probe 2′,7′-dichlorofluorescein diacetate (H_2_DCFDA) was employed to determine in vivo cytoplasmic ROS in day 14 worms grown on OP50 or LPJBC5, as described by Yoon et al. [52]. Briefly, the protein extract was prepared, and twenty-five micrograms of protein for each sample were dissolved in PBS to bring the final volume to 50 μL. The protein lysate (25 μg/sample) or PBS (control) were further mixed with 100 μL of 50 μM chloromethyl-H_2_DCFDA in PBS and transferred to wells of 96 wells black microplate. The plate was stored at 37 °C for 4 h, and fluorescence intensity was further measured using a multimode reader (Excitation-485, Emission-535) (Varioskan Flash, Thermo Scientific, USA). The experiment was performed with three biological replicates, and 100 worms per bacterial treated group were used for fluorescence measurement. The fluorescence intensity was normalized after subtracting the fluorescence intensity of the control (without worms).

### 2.24. Determination of Reactive Oxygen Production and Change in Transmembrane Potential of Mitochondria

MitoTracker^®^ Red CM-H2XRos reagent (M7513, Invitrogen, USA) was employed to determine mitochondrial ROS in day-14 worms grown on OP50 or LPJBC5 using a procedure described by Dilberger et al. [53]. Briefly, the worms were incubated in 5 μM MitoTracker^®^ Red CM-H2XRos reagent (M7513, Invitrogen, USA) at room temperature for 4 h. Then, the washed worms were visualized under a confocal microscope (excitation at 525 ± 45 nm, and emission at 595 ± 35 nm) [53]. The experiment was performed in triplicate, and 10 worms per bacterial-treated group were used for the measurement of fluorescence intensity.

The JC-1 staining dye (T3168, Invitrogen, USA) was used to determine the mitochondrial transmembrane potential in day-14 worms grown on OP50 or LPJBC5 by a previously described procedure Nakagawa et al. [12]. One hundred worms per group were used for fluorescence measurement, and the experiment was performed in replicates. The fluorescence intensity was normalized after subtracting the fluorescence intensity of the control (without worms).

### 2.25. Measurement of Intracellular Adenosine Triphosphate (ATP) Concertation

Roche ATP Bioluminescent HSII kit (Merck, Darmstadt, Germany) was used for bioluminescence-based detection of ATP concentrations as per the manufacturer’s instructions. Briefly, a standard curve of ATP (1 μM–1 × 10^−5^ μM) was prepared. Day-14 worms grown on OP50 or LPJBC5 were washed thrice with PBS, and protein extracts were prepared as described by Nakagawa et al. [12]. Bioluminescence of each sample or PBS (sample blank) was measured against the ATP standard in a multimode plate reader. The ATP concentration in each sample was calculated using a log-log plot of the standard curve and the value was expressed nmol of ATP/mg protein. One hundred worms per group were used to measure ATP concentration, and each experiment was performed in triplicate.

### 2.26. Quantification of Apoptosis by Tunnel-Assay

In Situ Cell Death Detection Kit, Fluorescein (Merck, Germany) was employed to detect and quantify apoptosis in day 17 worms grown on OP50 or LPJBC5. The procedure was followed as per the manufacturer’s instructions. One hundred worms per bacterial-treated group were used to detect fluorescence intensity, and each experiment was performed in triplicate.

### 2.27. Statistical Analysis

All represented data are results of independent three replicates, and every experiment was repeated twice. The data were represented as mean ± standard error mean (SEM) of three determinations. The OASIS 2 program was used for survival analysis of lifespan and other survival assays. The difference between the survival curve of worms was evaluated using the log-rank test. All statistical computation analysis was performed using SigmaPlot version 12.0 (San Jose, CA, USA). The statistical analysis between treatments/groups was carried out using one-way analysis of variance (ANOVA) and Student’s *t*-test. A Mann–Whitney U test was performed if the data were not normally distributed. Between two sets of data, the *p*-value of <0.05 was considered statistically significant.

## 3. Results

### 3.1. Molecular Taxonomic Characterisation of LPJBC5 and Persistence in In Vitro Gastrointestinal Conditions

The 16S rDNA sequence of LPJBC5 was submitted to the NCBI database (GenBank Acc. No. MG824976.1). A rooted phylogenetic tree of 16S rDNA gene sequence analysis of strain JBC5 showed similarity to the species *Lactobacillus plantarum*. Its closest relative species was found to be *Lactobacillus plantarum* strain CHE37 (MZ571407.1) with 100% sequence similarity (Figure S1). LPJBC5 was chosen based on the evaluation of probiotic attributes by in vitro analyses conducted according to the guidelines issued by the Indian Council of Medical Research and Department of Biotechnology, Govt. of India [54]. These parameters are resistance to acidic and bile conditions and the ability to colonize the intestinal epithelium cell line [54] (Table S3). Furthermore, *L. plantarum* species-specific PCR assay confirmed the presence of species-specific sequence, probiotic marker genes (encode bile salt hydrolase (*Lpbsh1*) and collagen-binding protein (*Lpcbp*)) and the antimicrobial plantaricin-biosynthetic gene (*pln*), with amplicon lengths 152, 975, 2174 and 231 bp (Figure S2A). All of these sequences have been submitted to the NCBI database (Table S4). The BLAST analysis of these sequences showed the closest pairwise sequence similarity to different *L. plantarum* strains (Table S4). The amplicon size of probiotic markers and plantaricin genes represented full-length *L. plantarum*-specific *bsh1*, *cbp*, and *pln* genes (Table S4).

### 3.2. LPJBC5 Increases Longevity and Slows the Development of Worms

**Figure 1:**
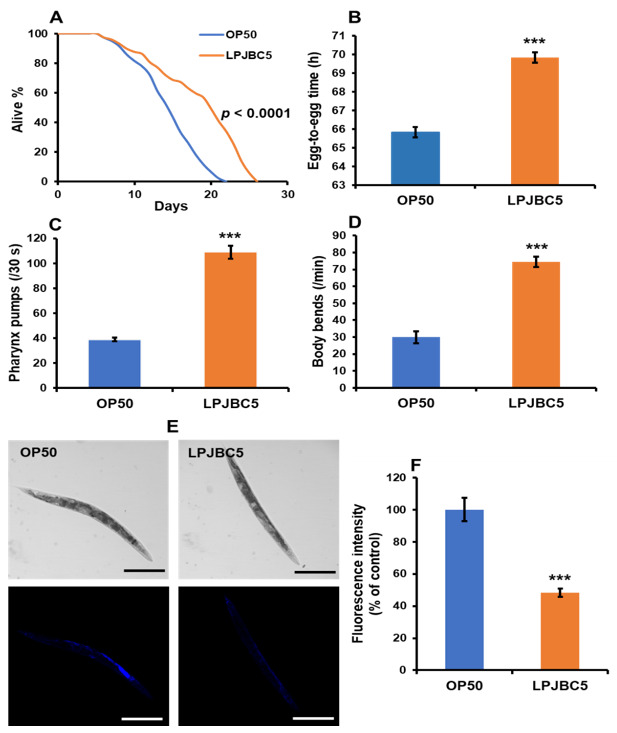
Feeding of probiotic *L. plantarum* JBC5 (LPJBC5) promotes longevity and age-related biomarkers of worms. (**A**) Lifespan assay on worms was performed after feeding with the bacteria, *E. coli* OP50 (OP50) or *L. plantarum* JBC5 (LPJBC5) (10^9^ CFU/mL). (**B**) Egg-to-egg time was used to assess the developmental rate after treatment with each bacterium (**C**,**D**) Pharyngeal pumping rate and locomotor activity were analyzed on the 14th day of bacterial treatments. (**E**,**F**) Aging biomarkers such as lipofuscin accumulation were assayed on the 14th day of bacterial treatment. Accumulation of lipofuscin was observed under a confocal microscope at 10× magnification (Scale bar, 250 μm). Error bars represent mean ± SEM. Treatment effects were compared using Student’s *t*-test (*** *p* < 0.001).

Feeding of LPJBC5 increased the lifespan of worms by 27.8% compared with those fed on standard bacterial food, *E. coli* OP50 (OP50) (*** *p* < 0.0001, log-rank test) (Figure 1A).

The effect of LPJBC5 on the developmental rate of worms showed that the developmental rate of LPJBC5-fed worms was significantly slower from eggs to reproductive adult stage (egg to egg) compared with OP50-fed (*** *p* < 0.001) (Figure 1B). Further, a significant decrease in body size of the LPJBC5-fed worms was observed compared with the OP50-fed (*** *p* < 0.001 on day 4 and 6; ** *p* < 0.01 on day 5 and 7) (Figure S2B).

### 3.3. LPJBC5 Is Efficiently Colonized into the Gut of Worms

The bacterial colony forming units (CFU) count increased with age in both LPJBC5 and OP50-fed worms (Figure S2C); however, the CFU count was found to be significantly higher in LPJBC5-fed worms on both 3rd and 8th days of incubation by 67.11% and 133.47% compared with OP50-fed worms (*** *p* < 0.001 for both 3rd and 8th day) (Figure S2C). Result showed that there was about a two-fold increase in colonization efficiency of LPJBC5 within the gut of worms on 8th day relative to OP50-fed worms.

### 3.4. Feeding of LPJBC5 Delayed Aging in Worms

The effect of feeding LPJBC5 on age-related biomarkers of worms, such as pharynx pumping, body bends, and lipofuscin accumulation, was investigated. Feeding of LPJBC5 significantly increased the pharyngeal pumping rates in worms by 179.47% higher on day-14 compared with OP50-fed (*** *p* < 0.001) (Figure 1C).

We next counted the number of body bends per minute. It was observed that the frequency of body bending was higher in LPJBC5-fed worms by 148.66% on day-14 compared with OP50-fed (*** *p* < 0.001) (Figure 1D). These results indicated that LPJBC5 increased lifespan and improved the quality of life in the later stage of life.

We also determined the accumulation of lipofuscin on 14-day-old worms fed with LPJBC5. The results showed that feeding LPJBC5 significantly reduced lipofuscin accumulation by 51.79% of worms compared with OP50-fed (*** *p* < 0.001) (Figure 1E,F).

### 3.5. Feeding of LPJBC5 Reduced the Accumulation of Fat in Worms

**Figure 2:**
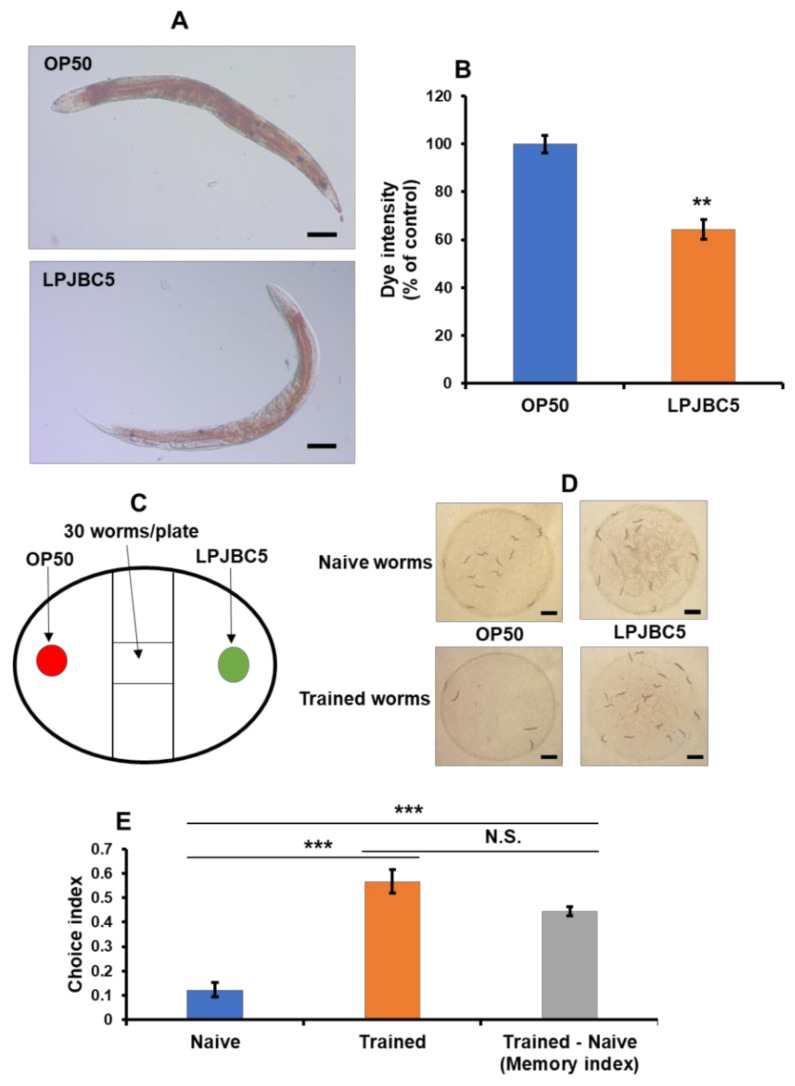
Treatment with LPJBC5 reduces lipid accumulation and improves the learning and memory of *C. elegans*. (**A**,**B**) Lipid accumulation was assayed on the 14th day of bacterial treatment. The images were observed at 10× (Scale bar, 100 μm). (**C**) The schematic diagram represents the experimental design. The experiment was performed on a 60 mm Petri-plate containing NGM medium for the binary choice assay. (**D**) Observation of results in naive and trained worms for binary choice assay. (Scale bar, 1 mm). (**E**) Choice index (CI) of food preference for naive and trained worms. Memory index (CI trained- CI naive) was calculated from the results of the binary choice assay. Error bars represent mean ± SEM. Treatment effects were compared using Student’s *t*-test (** *p* < 0.01 and *** *p* < 0.001).

The results showed that lipid droplet accumulation was reduced by 35.77% in the LPJBC5-fed worms compared with OP50-fed aged worms (** *p* < 0.01) (Figure 2A,B).

We next evaluated the number of progenies produced by young adult worms on days 1–5 after transferring to NGM plates seeded with LPJBC5 or OP50. The results showed no significant difference in the total number of offspring between LPJBC5- and OP50-fed worms (*p* > 0.05) (Figure S2D).

### 3.6. LPJBC5 Improved Learning and Memory in Worms

A binary choice assay was performed to examine the effect of LPJBC5 on the feeding behavior of worms. Firstly, the results showed no significant change in the feeding preference for LPJBC5-fed worms (CI = +0.12) compared with OP50-fed (*p* > 0.05) (Figure 2C–E). Then, we studied the effect of LPJBC5 on the learning ability of worms. Day-3 worms were trained on LPJBC5 for 4 h, and then a binary choice assay was performed. Trained worms showed greater feeding preference (CI = +0.56) for LPJBC5 compared with naive worms (CI = +0.12) (Trained over naive *** *p* < 0.001) (Figure 2D,E). 

Furthermore, the memory index of worms trained on LPJBC5 over naive worms (Trained–Naive) was calculated. A higher preference of trained worms for LPJBC5 over OP50 indicates a higher memory index. The results showed that the memory index of the worms fed on LPJBC5 was significantly higher over naive worms (CI = +0.44) (*** *p* < 0.001) (Figure 2E). These results indicated that feeding of LPJBC5 improved the learning and memory of worms.

### 3.7. Feeding of LPJBC5 Conferred Resistance against Abiotic and Biotic Stress Conditions

**Figure 3:**
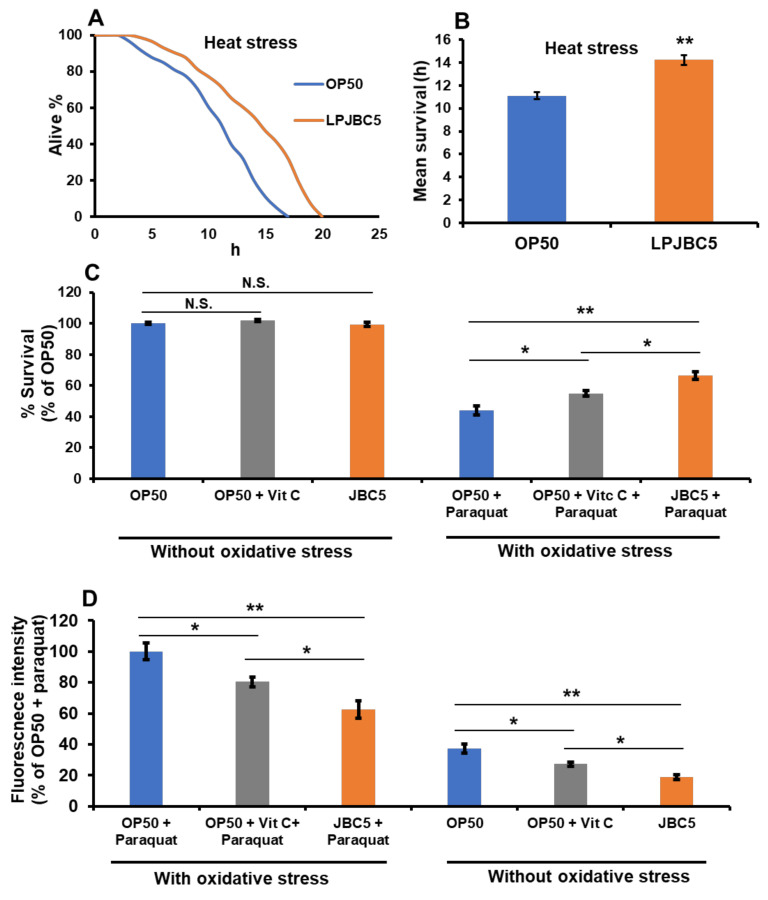
Treatment with LPJBC5 improved the resistance of worms against abiotic stresses. After treatment with LPJBC5, the survival of worms against heat stress at 35 °C (**A**,**B**), oxidative stress (paraquat-100 mM) as well as accumulation of lipofuscin level in presence and absence of oxidative stress (**C**,**D**) are presented. Bars represent mean ± SEM. Treatment effects were compared using Student’s *t*-test (* *p* < 0.05 and ** *p* < 0.01).

The results suggested that the survival of worms against thermal stress was 28.2% higher in LPJBC5-fed compared with OP50-fed worms (** *p* < 0.01) (Figure 3A,B). Next, the effect of LPJBC5 was evaluated on resistance against oxidative stress (100 mM paraquat) on worms. There was significant increase in the survival rate of LPJBC5-fed worms against oxidative stress compared with OP50-fed worms (** *p* < 0.01) (Figure 3C).

In addition, the results also showed a lower level of lipofuscin (a measure of senescence) in LPJBC5-fed worms compared with OP50-fed worms in both the presence and absence of oxidative stress conditions (** *p* < 0.01) (Figure 3D and Figure S3).

**Figure 4:**
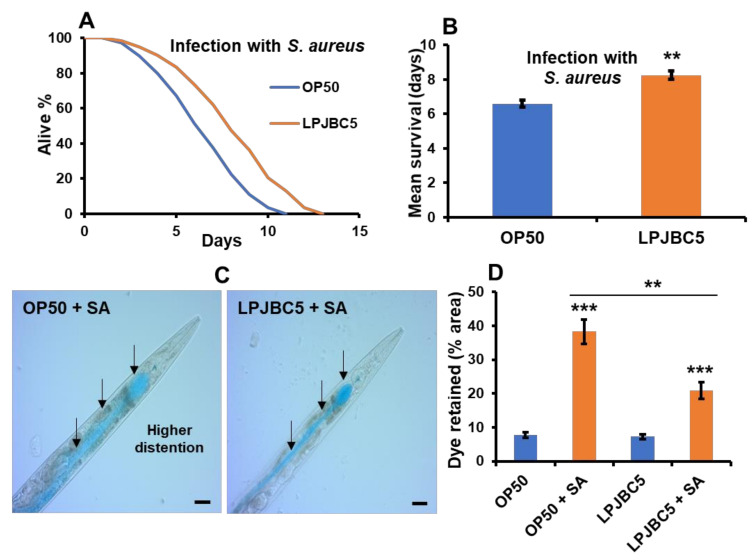
Treatment with LPJBC5 improved the resistance of worms against biotic stresses. The infection with pathogen *Staphylococcus aureus* (**A**,**B**). Feeding of LPJBC5 improved intestinal integrity of worms against pathogen *S. aureus* (SA) (**C**,**D**). The intestinal cavity was observed in treated groups (OP50 + SA and LPJBC5 + SA) under a compound microscope at 20× (**C**), and area (%) with blue food dye retained in the intestinal cavity per worm was calculated using ImageJ software (Scale bar, 20 μm) (**D**). Error bars represent mean ± SEM. Treatment effects were compared using Student’s *t*-test (** *p* < 0.01 and *** *p* < 0.001).

Next, we determined whether feeding of LPJBC5 increases the resistance of worms against pathogenic bacteria *S. aureus*. The results showed that feeding LPJBC5 increased survival by 25% against *S. aureus* compared with OP50-fed worms (** *p* < 0.01) (Figure 4A,B). Furthermore, the worms pre-cultured on LPJBC5 showed less distention of the intestinal lumen after pathogenic exposure compared with pre-cultured worms on OP50 (Mean ± SEM, 20.8 ± 1.38 for LPJBC5 vs. 38.26 +2.07 for pre-cultured on OP50) (** *p* < 0.01) (Figure 4C,D and Figure S4B).

### 3.8. Understanding the Pathway Involved in Pro-Longevity Effect of LPJBC5-Fed Worms

**Figure 5:**
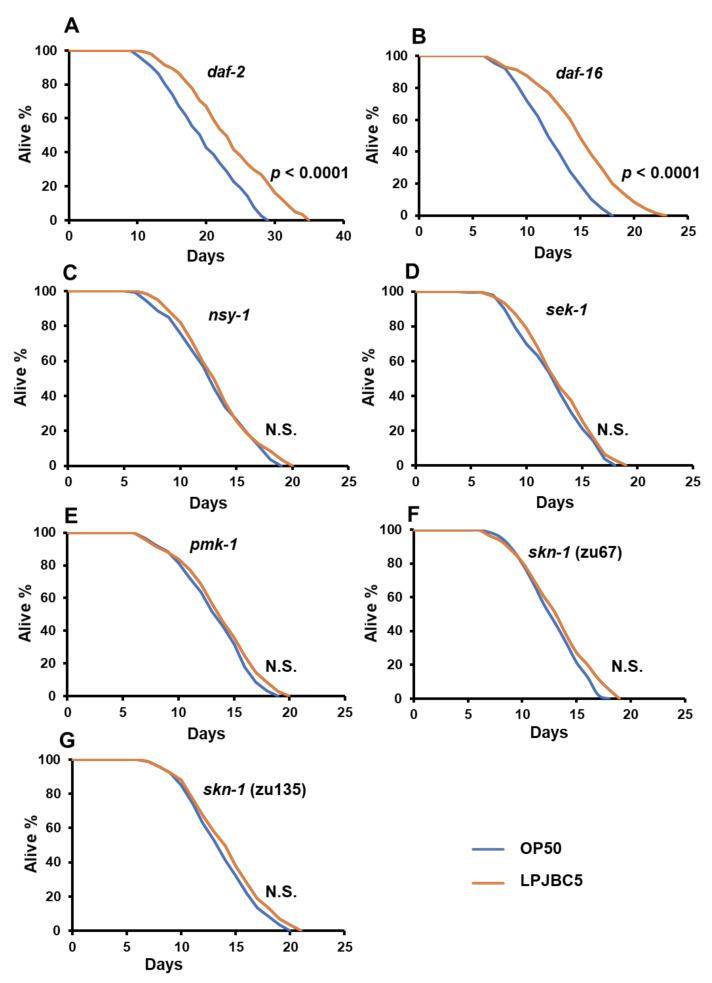
Elucidation of pathway involved in lifespan enhancement of worms by LPJBC5. Lifespan assays were performed with mutants (**A**) *daf-2* (e1368), (**B**) *daf-16* (mgDf50), (**C**) *nsy-1* (ag3), (**D**) *sek-1* (ag1), (**E**) *pmk-1* (km25), (**F**) *skn-1* (zu67) and (**G**) *skn-1* (zu135). Treatment effects were compared using log rank test.

We firstly investigated the role of DAF-2/DAF-16 mechanism involving two loss-of-function mutants *daf-2* (longer-lived) and *daf-16* (shorter-lived) of worms. The results showed that feeding of LPJBC5 to these two mutant worms extended their longevity (*daf-2*, *** *p* < 0.0001; *daf-16*, *** *p* < 0.0001, log-rank test) (Figure 5A,B, Table 1). This suggested that LPJBC5-induced longevity is not dependent on the involvement of DAF-2/DAF-16 pathway.

We further studied whether LPJBC5-induced longevity is due to the activation of p38 MAPK pathways. Feeding of LPJBC5 to three loss-of-function mutants of worms, i.e., *sek-1*, *nsy-1* and *pmk-1* failed to extend longevity (*p* > 0.05, log-rank test) (Figure 5C–E, Table 1). We next asked whether the downstream gene of p38 MAPK pathway, i.e., *skn-1* has a role in LPJBC5-induced longevity in worms. Two *skn-1* loss-of-function allele mutants, i.e., *skn-1* (zu67) and *skn-1* (zu135) were fed with LPJBC5, and determination of their lifespan showed that feeding of LPJBC5 were unable to extend longevity in either *skn-1* allele mutants (*p* > 0.05, log-rank test) (Figure 5F,G, Table 1).

### 3.9. Elucidating the Molecular Mechanisms of LPJBC5-Induced Healthy Aging in Worms

To further understand how LPJBC5 promoted healthy aging in worms, qRT-PCR was conducted to study the expression of important genes involved in longevity, fat accumulation, learning and memory, stress response, innate immunity, and intestinal integrity.

**Figure 6:**
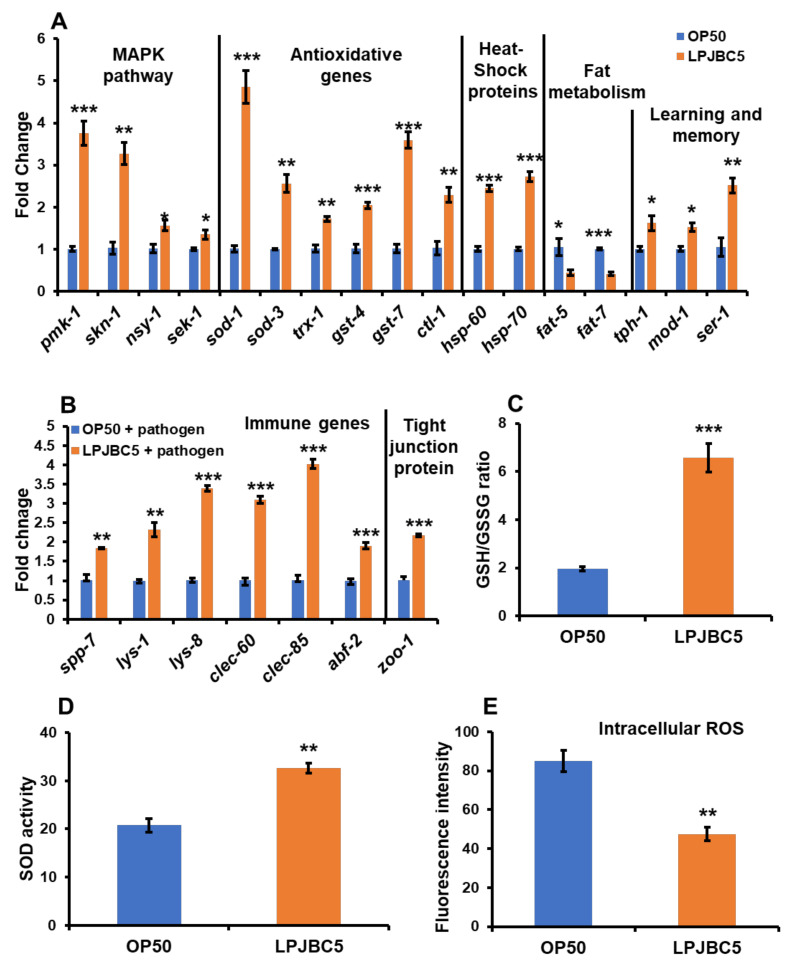
Expression of genes involved in longevity, stress resistance, fat accumulation, and learning and memory (**A**); genes involved in innate immunity and tight junction (ZOO-1) proteins in gut epithelium against infection with pathogen *S. aureus* (**B**). mRNA expression was normalized using the house-keeping gene *act-1*. Feeding of LPJBC5 improves GSH/GSSG ratio (**C**), SOD activity (**D**) and intracellular ROS levels (**E**) in worms compared with OP50-fed. Error bars represent mean ± SEM. Treatment effects were compared using Student’s *t*-test (* *p* < 0.05, ** *p* < 0.01 and *** *p* < 0.001).

First, the expression of key genes involved in p38 MAPK and DAF-2/DAF-16 genes was analyzed. The expression of *daf-2* and *daf-16* genes was unchanged in LPJBC5-fed worms in comparison with OP50-fed (*p* > 0.05 for both *daf-2* and *daf-16*) (Figure S4A). The expression of genes involved in p38 MAPK signaling, including *sek-1*, *nsy-1* and *pmk-1* were significantly upregulated in LPJBC5-fed worms (* *p* < 0.05 for *sek-1* and *nsy-1*, *** *p* < 0.001 for *pmk-1*) (Figure 6A and Figure S4A). We further studied whether the feeding of LPJBC5-induced p38 MAPK signaling activated further downstream target gene *skn-1*. It was observed that the expression of *skn-1* gene was increased by approximately two-fold in LPJBC5-fed worms in comparison with OP50-fed (Figure 6A) (** *p* < 0.01), but there was no significant change in the expression of the *skn-1b* gene, which is responsible for dietary restriction mediated longevity in worms (*p* > 0.05) (Figure S4A).

Second, the activated transcription factor SKN-1 activates the transcription of genes involved in phase-2 detoxification. There was increased expression of GSTs (*gst-4* and *gst-7*), catalases (*ctl-1* and *ctl2*), *trx-1* and SODs genes (*sod-1* and *sod-3*) in LPJBC5-fed worms in comparison with the expression of the same genes in OP50-fed worms (*** *p* < 0.001 for *sod-1*, *gst-4* and *gst-7*; ** *p* < 0.01 for *ctl-1*, *trx-1* and *sod-3*; * *p* < 0.05 for *ctl-2*) (Figure 6A and Figure S4A). Moreover, there was a two-fold increase in the expression of *hsp-60* and *hsp-70* genes in the LPJBC5-fed worms (*** *p* < 0.001 for *hsp-60* and *hsp-70*) (Figure 6A).

Third, the mRNA expression of genes encoding key substrates and enzymes of fat metabolism (*fat-5*, *fat-6,* and *fat-7*) was analyzed in LPJBC5-fed worms. Our results suggested that feeding of LPJBC5 significantly decreased the expression of *fat5* and *fat-7* (* *p* < 0.05 for *fat-5*; *** *p* < 0.001 for *fat-7*) (Figure 6A), although the expression of *fat-6* gene was unchanged in LPJBC5 in comparison with OP50-fed worms (*p* > 0.05 for *fat-6*) (Figure S4A).

Fourth, the role of serotonin signaling genes involved in the LPJBC5-induced learning ability of worms, namely *ser-1*, *mod-1*, and *tph-1*, was investigated [55]. Consistent with the results of learning and memory assays, the expression of all three serotonin-signaling pathway genes were significantly upregulated in LPJBC5-fed worms compared with OP50-fed (** *p* < 0.01 for *ser-1*, * *p* < 0.05 for *mod-1* and *tph-1*) (Figure 6A).

Fifth, the results showed upregulation of innate immunity genes such as saponin-like proteins (*spp-7*), lysozymes (*lys-1* and *lys-8*), CLEC genes (*clec-60* and *clec-85*) and antibacterial factor (ABF) (*abf-2* and *abf-3*) in LPJBC5-fed worms against infection with pathogen *S. aureus* compared with OP50-fed worms (*** *p* < 0.001 for *lys-1*, *lys-8*, *clec-60*, *clec-85* and *abf-2*; ** *p* < 0.01 for *spp-7* and *lys-1*; * *p* < 0.01 for *abf-3*) (Figure 6B and Figure S4A). No significant change in the expression of *abf-1* and *spp-1* genes was observed between the LPJBC5- and OP50-fed worm groups (*p* > 0.05) (Figure S4A).

Lastly, there was a two-fold increase in expression of tight junction protein zonula occludin *zoo-1* in worms pre-cultured on LPJBC5 after exposure against pathogen *S. aureus* compared with pre-cultured on OP50 (*** *p* < 0.001) (Figure 6B).

### 3.10. Feeding of LPJBC5 Reduced the Production of Reactive Oxygen Species

The effect of LPJBC5 was examined on the ratio of glutathione/glutathione disulfide (GSH/GSSG) in worms, which indicates an oxidative redox environment in worms. Our results suggested that feeding of LPJBC5 increased the ratio of GSH/GSSG by approximately three-fold on day 14 compared with OP50-fed worms (GSSG, ** *p* < 0.01; GSH, *** *p* < 0.001; GSH/GSSG *** *p* < 0.001) (Figure 6C and Figure S4C). Feeding of LPJBC5 also improved the SOD activity in day 14 worms by 57.35% compared with OP50-fed worms (** *p* < 0.01) (Figure 6D).

Next, 2′,7′-dichlorofluorescein diacetate (H_2_DCFDA) was employed to detect in vivo cytoplasmic ROS levels in worms. There was a 44.12% decrease in the fluorescence intensity in LPJBC5-fed worms in comparison with OP50-fed (** *p* < 0.01) (Figure 6E).

### 3.11. LPJBC5 Improved Mitochondrial Function in Worms

**Figure 7:**
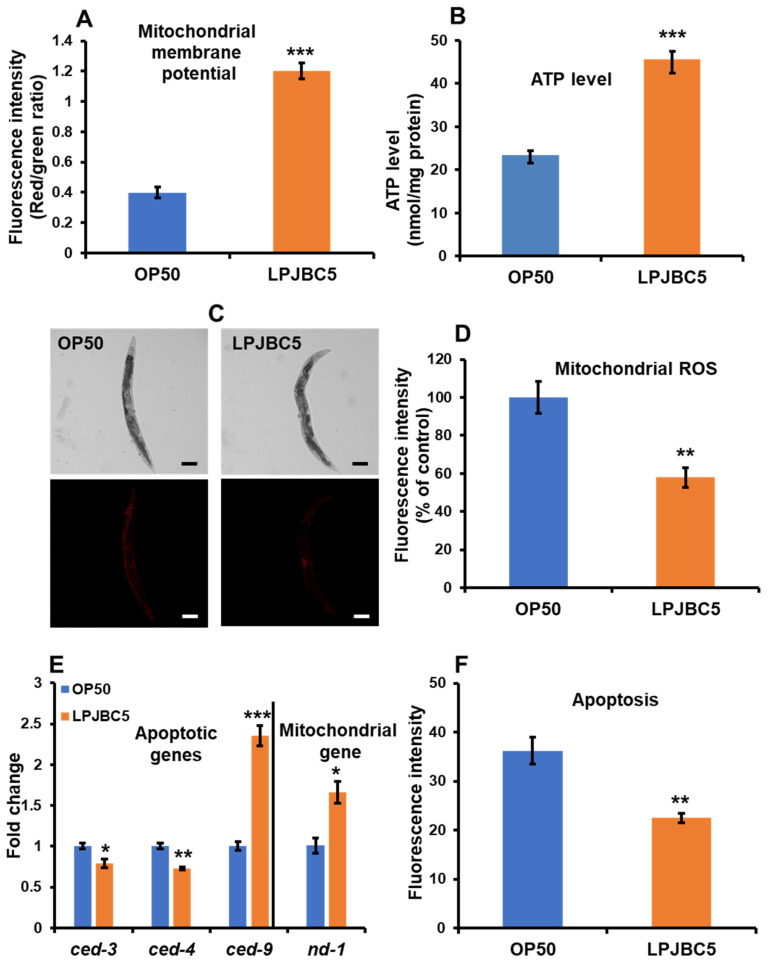
Treatment with LPJBC5 improves mitochondrial membrane potential (**A**), ATP synthesis (**B**), reduce mitochondrial ROS levels (**C**,**D**) (Scale bar, 100 μm). LPJBC5 treatment altered the expression of mitochondrial gene and genes involved in apoptosis (**E**). LPJBC5 treatment also supressed the rate of apoptosis (**F**) in worms. Error bars represent mean ± SEM. Treatment effects were compared using Student’s *t*-test (* *p* < 0.05, ** *p* < 0.01 and *** *p* < 0.001).

The JC-1 dye was used to investigate the effect of LPJBC5 on the transmembrane potential of mitochondria. JC-1 penetrates the non-apoptotic cells forming aggregates and shows red fluorescence, whereas JC-1 present as monomers in the apoptotic cells shows green fluorescence. Our results suggested that LPJBC5-fed worms showed a significantly lower level of green fluorescence and a higher level of red fluorescence intensity compared with OP50-fed (*** *p* < 0.001 for both red and green fluorescence) (Figure S4D). Additionally, the ratio of red/green fluorescence intensity was three-fold higher in LPJBC5-fed worms compared with OP50-fed (*** *p* < 0.001) (Figure 7A).

We further investigated the effect of LPJBC5 on synthesis of ATP levels in worms. LPJBC5-fed worms showed a 95.65% higher level of ATP in day-14 worms compared with OP50-fed worms under same experimental conditions (*** *p* < 0.001) (Figure 7B). Next, LPJBC5-fed worms were stained with MitoTracker Red CMXRos to analyze the level of mitochondrial ROS. There was a 42.09% reduction in mitochondrial ROS in LPJBC5-fed worms compared with OP50-fed (** *p* < 0.01) (Figure 7C,D).

In addition, we also studied the change in the expression of mitochondrial DNA (mtDNA) encoded gene *nd-1*. The results showed increased expression of *nd-1* in LPJBC5-fed worms in comparison with OP50-fed worms (* *p* < 0.05) (Figure 7E). Overall, the feeding of LPJBC5 significantly improved the functioning of mitochondria in the older age of worms.

### 3.12. LPJBC5 Retards Programmed Cell Death in Worms

Our results showed that apoptotic cell death was reduced by 37.74% in LPJBC5-fed worms compared with OP50-fed (** *p* < 0.01) (Figure 7F). In support, our qRT-PCR results showed increased expression of anti-apoptotic gene *ced-9*, whereas expression of pro-apoptotic genes (*ced-3* and *ced-4*) was significantly reduced in LPJBC5-fed compared with OP50-fed worms (*** *p* < 0.001 for *ced-9*; * *p* < 0.05 for *ced-3*; ** *p* < 0.01 for *ced-4*) (Figure 7E).

## 4. Discussion

The rapid progress in medical science has led to increasing lifespans. A recent report of the United Nations predicts that one in every six individuals will be older than the age of 65 by 2050 compared with one in every eleven in 2019 [3]. The increased life expectancy is not proportional to the quality of life in the elderly [3]. Further, increased lifespan has been linked with a higher risk of age-associated diseases, such as cancer, obesity, diabetes, cardiovascular disease, and neurodegenerative disorder [56]. It results in a significant burden on the global economy, as public expenditure has increased substantially with increased life expectancy [57]. Based on the recent developments in the gut microbiome and fermented foods, scientists are recollecting to understand many ethnic foods and their microbes for human health. In this study, a potential probiotic bacterium *L. plantarum* strain JBC5 isolated from curd was evaluated for its ability to enhance lifespan and promote healthy aging in worms.

In our study, LPJBC5 persisted in in vitro simulated gastrointestinal tests and could tolerate low pH (pH 1.0, 3.0, and 7.0) and bile acid (0.3–1.0%) without a significant decrease in viability (Table S3). Our finding corresponds to previous studies and suggests that LPJBC5 showed good survival ability to simulated gastric juice and bile acid [58]. Secondly, LPJBC5 was also able to adhere to intestinal cell line HT-29 (Table S3). Furthermore, the potential probiotic genes (collagen-binding protein and bile salt hydrolase) and antimicrobial plantaricin gene were confirmed in LPJBC5. The occurrence of collage-binding protein in LPJBC5 supported its candidature to colonize the host’s intestine efficiently. This hints that LPJBC5 can tolerate gastrointestinal conditions (stomach, small and large intestine) in humans and colonize the intestinal mucosa.

Our results suggest that both live and dead probiotic LPJBC5 could extend the lifespan of worms. Aging is a multifactorial phenomenon regulated by diverse signaling mechanisms; few mechanisms were important in anti-aging process [5]. Several mechanisms, for example, p38 MAPK and DAF-2/DAF-16 pathways, have been identified to extend longevity in worms [59]. The mechanisms of longevity may also regulate the conserved genes involved in stress resistance, anti-oxidative pathways, immunity, and metabolism [59]. In this study, it was observed that in the p38 MAPK pathway mutant worms (*sek-1*, *nsy-1*, and *pmk-1*), LPJBC5 was unable to extend longevity. The activated p38 MAPK pathway may also induce the expression of downstream transcription factor SKN-1, which regulates several processes, such as detoxification, immune response, and metabolism. We confirmed that the feeding of LPJBC5 to allelic *skn-1* mutant worms did not extend longevity. In contrast, the feeding of LPJBC5 to mutants of DAF-2/DAF-16 pathways (*daf-2* and *daf-16*) extended the longevity. Our qRT-PCR results also increased the expression of genes involved in p38 MAPK pathway and its downstream target *skn-1*. Nevertheless, there was no significant change in gene expression involved in the DAF-2/DAF-16 pathway. Thus, we conclude that the feeding LPJBC5 activated *skn-1* through the p38 MAPK signaling pathway to extend longevity in worms. This result is in accordance to the recent study by Zhou et al. [60].

Previous reports suggested that longevity is not straightforward, and demonstrated the fundamental relationship between slowed development and longevity [61]. Our study found that feeding of LPJBC5 compared with feeding of OP50 slowed the developmental rate and reduced the body size in worms. The smaller body size of worms is often linked with increased lifespan, which is considered to be as a result of slowed developmental rate [41,62]. Generally, longevity is accompanied by positively affecting the aging biomarkers in worms, including the rate of pharynx pumping, locomotory activity, and reduction in lipofuscin accumulation [63]. We found that feeding of LPJBC5 improved the rate of pharyngeal pumping and the number of body bends and showed a decrease in lipofuscin accumulation in worms. These results suggested that feeding of LPJBC5 effectively improved the quality of life in the elderly.

Emerging pieces of evidence suggest that fat metabolism is tightly regulated in the aging process [64]. In worms, the RNA interference (RNAi) and mutant studies have found that silencing fat genes, including *fat-6*, *fat-5*, and *fat-7*, decreased the fat accumulation under normal physiological conditions [65,66]. The over-expression of the *fat-7* gene in worms promoted fat accumulation and reduced the lifespan of transgenic worms [67]. Our results suggested that feeding LPJBC5 reduced the fat storage in worms compared with OP50-fed ones. In support, we found that LPJBC5 reduced the expression of genes encoding key substrates and enzymes of fat metabolism (*fat-5* and *fat-7*) compared with OP50-fed worms.

Diet can affect behavior, ranging from feeding, sensory, learning, and memory [10]. Our study demonstrated that adult worms had no feeding preference for LPJBC5 over OP50. Moreover, we trained worms on LPJBC5 to assess its role in learning and memory. We observed a higher preference of LPJBC5-trained worms for LPJBC5 over OP50, suggesting worms pre-cultured on LPJBC5 improved their learning and memory for LPJBC5 to choose LPJBC5 over OP50 compared with naive worms precultured on OP50. Previous studies suggest that serotonin signaling modulates the behavior of worms [68]. Our qRT-PCR results indicated that feeding of LPJBC5 improved the expression of genes involved in serotonin signaling, thus suggesting a link for communication between the central nervous system and gastrointestinal tract. The present study only provided proof that pre-conditioned or trained worms on LPJBC5 improved learning and memory, but furthermore, a mechanistic approach is needed to comment on its role in the gut–brain axis.

In mammals, the p38 MAPK pathway activates the transcription of genes involved in inflammatory cytokines and antimicrobials against exposure to lipopolysaccharide (LPS) [69,70,71]. PMK-1, a vital component of p38 MAPK, is important in in innate immune defences of worms against pathogens [72]. Previous studies have suggested that the gut pathogen *S. aureus* primarily colonizes and causes distention of the intestine of worms, then kills the worms in 5–7 days [73]. We observed that LPJBC5 improved the survival rate of worms against infection with *S. aureus*. We confirmed that feeding of LPJBC5 over-expressed the *pmk-1*.

Additionally, LPJBC5 also increased the expression of genes for antimicrobial proteins and peptides (*lys-1*, *lys-8*, *clec-60*, *clec-85*, *abf-2* and *spp-7*). The innate immunity of *C. elegans* involves antimicrobial peptides (defensin-like peptides) and proteins (lysozymes, caenacins, saponin like proteins, C-type lectin domain-containing (CLEC) proteins) [11]. Lysozymes cleaves the chemical bond between *N*-acetylmuramic (NAM) and N-acetylglucosamine (NAG) acid. In a previous study, lysozyme expression in worms, including lys-1 and lys-8, limited the accumulation of the pathogen (*Serratia marcescens*) in their intestine [74]. Saponin-like protein (spp.) are N-terminal small lysosomal proteins containing more than 35 amino acid residues which form pores into the pathogenic membranes. Silencing of Saponin-like protein genes decreased the bacterial load in their intestine [75]. CLEC proteins are carbohydrate-binding protein domain known to perform several functions, especially cell to cell adhesion and immune response against pathogen. The study conducted by Miltsch et al. [76] reported that knockdown of clec genes (*clec-39* and *clec-49*) reduced survivability of *C. elegans* against pathogen *Serratia marcescens* [76]. Antibacterial factor (*abf*) or defensin-like peptides are cysteine-rich cationic proteins, which perform important role in creating voltage-dependent channel in cell membrane of pathogen and disrupts it. Knockdown of *abf-2* by RNAi decreased the survival of *C. elegans* by allowing the increase in microbial growth (*Salmonella typhimurium*) in the intestine [77].

In support, we observed that LPJBC5 counteracted the disruption of intestinal epithelium caused by the pathogen *S. aureus*. The impaired intestinal barrier is characterized by the loss of the epithelial tight junction. In particular, zonula occludin ZOO-1 (human ZO-1 homolog) is a tight junction protein and acts as a representative marker for tight junction integrity [78]. Mechanistically, we found that feeding of LPJBC5 increased the expression of a gene encoding for tight junction proteins, *zoo-1*. Some other probiotic bacteria, such as *Bifidobacterium bifidium* have also been shown to strengthen the tight junction of intestinal epithelium in epithelium monolayer models [79].

Previous research suggested that ROS level increases with age and plays a role in more than 60 diseases, such as rheumatoid arthritis, neurodegenerative diseases, and gastrointestinal diseases [80]. Notably, the cellular redox state is commonly determined by the level of SOD activity and GSH/GSSG ratio [81]. It was observed that there was an increase in SOD activity and GSH/GSSG ratio in LPJBC5-fed compared with OP50-fed worms, thereby reducing the accumulation of ROS levels. In addition, we found that feeding of LPJBC5 increased the overall survival rate of worms under oxidative and heat-stressed conditions. In support, the feeding of LPJBC5 upregulated the expression of essential anti-oxidative genes (*sod-1*, *sod-3, ctl-1*, *ctl2, gst-4*, *gst-7*, and *trx-1*) as well as genes encoding heat-shock proteins (HSPs) (*hsp-60* and *hsp-70*). The over-expressed HSPs may counteract the protein misfolding due to changes in cellular redox state [82,83].

Mitochondria are the primary source for the production of ROS within cells. The mitochondrial free radical theory of aging (MFRTA) proposed that an imbalance between ROS and cellular defense mechanisms may induce mitochondrial dysfunction, resulting in the production of more ROS, which further leads to aging and cell death [84]. The decline in mitochondrial function has been correlated with developing different pathologies such as metabolic disorders, type-2 diabetes, and cancer [85]. We found that feeding LPJBC5 decreased the production of mitochondrial ROS and improved mitochondrial membrane potential compared with OP50-fed worms. The mitochondrial electrochemical gradient is a crucial bioenergetic parameter to access the mitochondrial function that further controls ATP synthesis. In addition, the feeding of LPJBC5 also upregulated the expression of mitochondrial DNA (mtDNA)-encoded NADH-ubiquinone oxidoreductase chain 1 (*nd-1*). As a result, LPJBC5 increased the level of ATP synthesis compared with OP50-fed worms.

Notably, the higher mitochondrial dysfunction is often correlated with an increased apoptosis rate or programmed cell death [86]. We also confirmed that LPJBC5 reduced apoptotic cell death in day-17 worms compared with OP50-fed. In support of this observation, the qRT-PCR results showed that pro-apoptotic gene expression was significantly reduced, whereas anti-apoptotic gene expression was increased in LPJBC5-fed worms. This result suggests that LPJBC5 effectively improved mitochondrial function and reduced the rate of apoptosis in worms.

## 5. Conclusions

**Figure 8:**
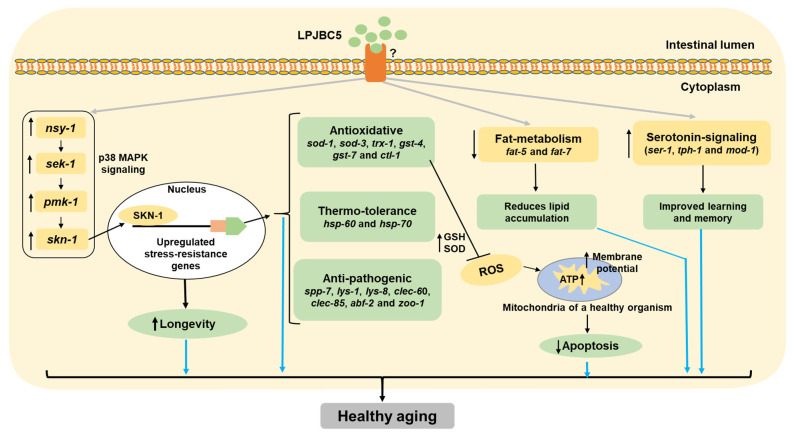
Proposed mechanisms of healthy aging induced by LPJBC5 in worms. Feeding of LPJBC5 activates p38 MAPK signaling cascade (*nsy-1*-*sek-1*-*pmk-1*), which further activates the downstream SKN-1 transcription factor. The activated SKN-1 further activates the transcription of phase-II detoxification genes or antioxidative genes. These upregulated antioxidative genes extend longevity, increasing the resistance against stress (oxidative and heat) and infection against pathogen *S. aureus*. The improved antioxidative machinery increases mitochondrial function and ATP synthesis, thereby reducing apoptosis in worms. Feeding of LPJBC5 also reduces fat storage through downregulation of genes encoding key substrates and enzymes of fat metabolism, including *fat-5* and *fat-7*. In addition, the learning and memory of worms were enhanced in LPJBC5-fed worms by upregulating the genes involved in serotonin signaling. Therefore, feeding of LPJBC5 activates the *skn-1* induced pathways to extend longevity, stress resistance, and immunity, and reduces other age-associated pathologies.

Over a century ago, Dr. Elie Metchnikoff hypothesized that lactic acid bacteria promote longevity and healthy aging in humans because Bulgarians consumed a sufficient amount of yogurt in their diet. Recent research suggests that similar longevity and aging mechanisms operate in *C. elegans*. Our study suggests that feeding of LPJBC5 activates the p38 MAPK pathway and its downstream targets to enhance longevity by improving stress resistance and immunity and other age-associated pathologies in worms (Figure 8). We believe that LPJBC5 has great potential to promote longevity and healthy aging, and prevent several age-associated disorders in mammals; thus, it should be promoted as a next-generation probiotic.

## Figures and Tables

**Table 1:** The mean lifespan of wild-type and mutant strains of *C. elegans* fed with OP50 or probiotic LPJBC5. N.S. corresponds to the non-significant *p* value.

Strain	Bacterial Source	Mean Life Span ± SEM (Days)	Total Worms (150) = Dead/Censored	*p*-Value (*L. plantarum* JBC5 versus *E. coli* OP50)
N2 (wild-type)	OP50	14.56 ± 0.34	138/12	*p* < 0.0001 (***)
	LPJBC5	18.61 ± 0.48	143/7	
*daf-2* (e1368)	OP50	19.67 ± 0.45	141/9	*p* < 0.0001 (***)
	LPJBC5	23.48 ± 0.51	142/8	
*daf-16* (mgDf50)	OP50	12.54 ± 0.24	139/11	*p* < 0.0001 (***)
	LPJBC5	15.22 ± 0.32	141/9	
*nsy-1* (ag3)	OP50	13.04 ± 0.28	134/16	*p* > 0.05 (N.S.)
	LPJBC5	13.50 ± 0.26	137/13	
*sek-1* (ag1)	OP50	12.72 ± 0.27	138/12	*p* > 0.05 (N.S.)
	LPJBC5	13.13 ± 0.22	140/10	
*pmk-1* (km25)	OP50	13.51 ± 0.25	142/8	*p* > 0.05 (N.S.)
	LPJBC5	13.94 ± 0.30	143/7	
*skn-1* (zu67)	OP50	13.03 ± 0.23	140/10	*p* > 0.05 (N.S.)
	LPJBC5	13.49 ± 0.27	142/8	
*skn-1* (zu135)	OP50	13.83 ± 0.19	139/11	*p* > 0.05 (N.S.)
	LPJBC5	14.37 ± 0.28	136/14	

## Data Availability

All relevant data are provided in the manuscript and supplementary file. Additionally, the DNA sequences including species-specific sequence (*Plantarum*), probiotic marker genes (encode bile salt hydrolase (*Lpbsh1*) and collagen-binding protein (*Lpcbp*)) and the antimicrobial plantaricin-biosynthetic gene (*pln*) of LPJBC5 used in our study are publicly available in National Center for Biotechnology Information, Bethesda, USA (NCBI) database. The raw fastq files of these sequences can be accessed through the GenBank accession number MW846636- MW846639 in the link https://www.ncbi.nlm.nih.gov/nuccore/.

## Supplementary Materials

The following supporting information can be downloaded at: https://www.mdpi.com/article/10.3390/antiox11020268/s1. Figure S1: Neighbour-joining tree was based on 16S rRNA gene sequences (1297 bases) of *L. plantarum* JBC5 (Accession no.: MG824976.1) and other related species of *Lactobacillus*. Bootstrap values (expressed as percentages of 1000 replications) greater than 60% are given at nodes. GenBank accession number are provided in the parentheses of each strain. *Enterococcus faecium* ATCC 19434 (Accession no.: DQ411813.1) was used as an outgroup. The evolutionary distances were computed using the Kimura 2-parameter method in MEGA 7 software, representing the number of base substitutions per site. The bar represents 0.01 substitutions per nucleotide position. Figure S2: A. Figure S2 A An agarose gel image of PCR amplified products of *Lactobacillus plantarum* specific antimicrobial plantaricin-biosynthetic gene (*Pln*) PlnF/R (lane 1: ~231 bp) (Acc. no.: MW846638), species-specific segment PlantarumF/R (lane 2: ~152 bp) (Acc. no.: MW846639), probiotic marker genes (i.e., bile salt hydrolase (*Lpbsh1*) (lane 3: ~975 bp) (Acc. no.: MW846636) and collagen-binding protein (*Lpcbp*) (lane 4: ~2174) (Acc. no.: MW846637) B. Effect of LPJBC5 on the body size of worms (*** *p* < 0.0001, log-rank test). C and D. The colonization efficiency and brood size were analyzed after feeding OP50 or LPJBC5. Error bars represent mean ± SEM. Treatment effects were compared using Student’s *t*-test (** *p* < 0.01 and *** *p* < 0.001). Figure S3: Accumulation of lipofuscin (i.e. a measure of senescence) with and without exposure to oxidative stress (100 mM paraquat) was observed under a confocal microscope at 10× magnification (Scale bar, 100 μm). Figure S4: A. qRT-PCR analysis on the expression of genes involved in longevity, stress resistance, immunity, and fat accumulation. B. The intestinal integrity of worms was observed in control groups OP50 and LPJBC5 under a compound microscope at 20× (Scale bar, 20 μm). The feeding of LPJBC5 improves GSH, reduces GSSG level (C), improves mitochondrial membrane potential in worms (D). Error bars represent mean ± SEM. Treatment effects were compared using Student’s *t*-test (* *p* < 0.05, ** *p* < 0.01 and *** *p* < 0.001). Table S1: Primers used to characterize the LPJBC5. Table S2: Primer sets used for qRT-PCR analysis. Table S3: Survival and adhesion of LPJBC5 under in vitro simulated gastro-intestinal conditions. Table S4: Closest gene homologs of LPJBC5 in the NCBI database.

## References

[1] Bixby R.L. (2020). Impacts of aging on the federal budget and economy: A cross-cutting challenge. Public Policy Aging Rep..

[2] Michel J.-P., Sadana R. (2017). “Healthy aging” concepts and measures. J. Am. Med. Dir. Assoc..

[3] United Nations (2019). World Population Ageing 2019: Highlights (ST/ESA/SER. A/430).

[4] Jin K., Simpkins J.W., Ji X., Leis M., Stambler I. (2015). The critical need to promote research of aging and aging-related diseases to improve health and longevity of the elderly population. Aging Dis..

[5] Fontana L., Partridge L. (2015). Promoting health and longevity through diet: From model organisms to humans. Cell.

[6] Brooks-Wilson A.R. (2013). Genetics of healthy aging and longevity. Hum. Genet..

[7] Ros M., Carrascosa J.M. (2020). Current nutritional and pharmacological anti-aging interventions. Biochim. Biophys. Acta-Mol. Basis Dis..

[8] Han B., Sivaramakrishnan P., Lin C.-C.J., Neve I.A., He J., Tay L.W.R., Sowa J.N., Sizovs A., Du G., Wang J. (2017). Microbial genetic composition tunes host longevity. Cell.

[9] Van Der Lugt B., Van Beek A.A., Aalvink S., Meijer B., Sovran B., Vermeij W.P., Brandt R.M., De Vos W.M., Savelkoul H.F., Steegenga W.T. (2019). *Akkermansia muciniphila* ameliorates the age-related decline in colonic mucus thickness and attenuates immune activation in accelerated aging *Ercc1^−/Δ7^* mice. Immun. Ageing.

[10] Xiao R., Chun L., Ronan E.A., Friedman D.I., Liu J., Xu X.S. (2015). RNAi interrogation of dietary modulation of development, metabolism, behavior, and aging in *C. elegans*. Cell Rep..

[11] Kumar A., Baruah A., Tomioka M., Iino Y., Kalita M.C., Khan M. (2020). *Caenorhabditis elegans*: A model to understand host–microbe interactions. Cell. Mol. Life Sci..

[12] Nakagawa H., Shiozaki T., Kobatake E., Hosoya T., Moriya T., Sakai F., Taru H., Miyazaki T. (2016). Effects and mechanisms of prolongevity induced by *Lactobacillus gasseri* SBT2055 in *Caenorhabditis elegans*. Aging Cell.

[13] Lee J., Yun H.S., Cho K.W., Oh S., Kim S.H., Chun T., Kim B., Whang K.Y. (2011). Evaluation of probiotic characteristics of newly isolated *Lactobacillus* spp.: Immune modulation and longevity. Int. J. Food Microbiol..

[14] Ikeda T., Yasui C., Hoshino K., Arikawa K., Nishikawa Y. (2007). Influence of lactic acid bacteria on longevity of *Caenorhabditis elegans* and host defense against *Salmonella enterica* serovar enteritidis. Appl. Environ. Microbiol..

[15] Fontana L., Partridge L., Longo V.D. (2010). Extending healthy life span—From yeast to humans. Science.

[16] Sánchez B., Delgado S., Blanco-Míguez A., Lourenço A., Gueimonde M., Margolles A. (2017). Probiotics, gut microbiota, and their influence on host health and disease. Mol. Nutr. Food Res..

[17] Metchnikoff E., Mitchell P.C. (1907). The Prolongation of Life: Optimistic Studies.

[18] Jomehzadeh N., Javaherizadeh H., Amin M., Saki M., Al-Ouqaili M.T., Hamidi H., Seyedmahmoudi M., Gorjian Z. (2020). Isolation and identification of potential probiotic *Lactobacillus* species from feces of infants in southwest Iran. Int. J. Infect. Dis..

[19] Patel P.J., Singh S.K., Panaich S., Cardozo L. (2014). The aging gut and the role of prebiotics, probiotics, and synbiotics: A review. J. Clin. Gerontol. Geriatr..

[20] Lew L., Hor Y., Jaafar M., Lau A., Ong J., Chuah L., Yap K., Azzam G., Azlan A., Liong M. (2019). Lactobacilli modulated AMPK activity and prevented telomere shortening in ageing rats. Benef. Microbes.

[21] Nam B., Kim S.A., Nam W., Jeung W.H., Park S.-D., Lee J.-L., Sim J.-H., Jang S.S. (2019). *Lactobacillus plantarum* HY7714 restores TNF-α induced defects on tight junctions. Prev. Nutr. Food Sci..

[22] Poupet C., Saraoui T., Veisseire P., Bonnet M., Dausset C., Gachinat M., Camarès O., Chassard C., Nivoliez A., Bornes S. (2019). *Lactobacillus rhamnosus* Lcr35 as an effective treatment for preventing *Candida albicans* infection in the invertebrate model *Caenorhabditis elegans*: First mechanistic insights. PLoS ONE.

[23] Dinić M., Herholz M., Kačarević U., Radojević D., Novović K., Đokić J., Trifunović A., Golić N. (2021). Host–commensal interaction promotes health and lifespan in *Caenorhabditis elegans* through the activation of HLH-30/TFEB-mediated autophagy. Aging.

[24] Yu X., Li S., Yang D., Qiu L., Wu Y., Wang D., Shah N.P., Xu F., Wei H. (2016). A novel strain of *Lactobacillus mucosae* isolated from a Gaotian villager improves in vitro and in vivo antioxidant as well as biological properties in D-galactose-induced aging mice. J. Dairy Sci..

[25] Park M.R., Shin M., Mun D., Jeong S.-Y., Jeong D.-Y., Song M., Ko G., Unno T., Kim Y., Oh S. (2020). Probiotic *Lactobacillus fermentum* strain JDFM216 improves cognitive behavior and modulates immune response with gut microbiota. Sci. Rep..

[26] Schifano E., Zinno P., Guantario B., Roselli M., Marcoccia S., Devirgiliis C., Uccelletti D. (2019). The foodborne strain *Lactobacillus fermentum* MBC2 triggers pept-1-dependent pro-longevity effects in *Caenorhabditis elegans*. Microorganisms.

[27] Ahmadi S., Wang S., Nagpal R., Wang B., Jain S., Razazan A., Mishra S.P., Zhu X., Wang Z., Kavanagh K. (2020). A human-origin probiotic cocktail ameliorates aging-related leaky gut and inflammation via modulating the microbiota/taurine/tight junction axis. JCI Insight.

[28] Park M., Park E.-J., Kim S.-H., Lee H.-J. (2021). *Lactobacillus plantarum* ATG-K2 and ATG-K6 Ameliorates High-Fat with High-Fructose Induced Intestinal Inflammation. Int. J. Mol. Sci..

[29] Zaydi A., Lew L.-C., Hor Y.-Y., Jaafar M., Chuah L.-O., Yap K.-P., Azlan A., Azzam G., Liong M.-T. (2020). *Lactobacillus plantarum* DR7 improved brain health in aging rats via the serotonin, inflammatory and apoptosis pathways. Benef. Microbes.

[30] Ong J.S., Taylor T.D., Yong C.C., Khoo B.Y., Sasidharan S., Choi S.B., Ohno H., Liong M.T. (2020). *Lactobacillus plantarum* USM8613 aids in wound healing and suppresses *Staphylococcus aureus* infection at wound sites. Probiotics Antimicrob. Proteins.

[31] Zhao J., Tian F., Yan S., Zhai Q., Zhang H., Chen W. (2018). *Lactobacillus plantarum* CCFM10 alleviating oxidative stress and restoring the gut microbiota in d-galactose-induced aging mice. Food Funct..

[32] Peng X., Meng J., Chi T., Liu P., Man C., Liu S., Guo Y., Jiang Y. (2014). *Lactobacillus plantarum* NDC 75017 alleviates the learning and memory ability in aging rats by reducing mitochondrial dysfunction. Exp. Ther. Med..

[33] Joishy T.K., Dehingia M., Khan M.R. (2019). Bacterial diversity and metabolite profiles of curd prepared by natural fermentation of raw milk and back sloping of boiled milk. World J. Microbiol. Biotechnol..

[34] Kumar R., Grover S., Batish V.K. (2011). Molecular identification and typing of putative probiotic indigenous *Lactobacillus plantarum* strain Lp91 of human origin by specific primed-PCR assays. Probiotics Antimicrob. Proteins.

[35] Khemariya P., Singh S., Jaiswal N., Chaurasia S. (2016). Isolation and identification of *Lactobacillus plantarum* from vegetable samples. Food Biotechnol..

[36] Kim E., Yang S.-M., Lim B., Park S.H., Rackerby B., Kim H.-Y. (2020). Design of PCR assays to specifically detect and identify 37 *Lactobacillus* species in a single 96 well plate. BMC Microbiol..

[37] Kumar S., Stecher G., Tamura K. (2016). MEGA7: Molecular evolutionary genetics analysis version 7.0 for bigger datasets. Mol. Biol. Evol..

[38] Conway P., Gorbach S., Goldin B. (1987). Survival of lactic acid bacteria in the human stomach and adhesion to intestinal cells. J. Dairy Sci..

[39] Vinderola C.G., Reinheimer J.A. (2003). Lactic acid starter and probiotic bacteria: A comparative “in vitro” study of probiotic characteristics and biological barrier resistance. Food Res. Int..

[40] Ayeni F.A., Sánchez B., Adeniyi B.A., Clara G., Margolles A., Ruas-Madiedo P. (2011). Evaluation of the functional potential of *Weissella* and *Lactobacillus* isolates obtained from Nigerian traditional fermented foods and cow’s intestine. Int. J. Food Microbiol..

[41] Kwon G., Lee J., Lim Y.-H. (2016). Dairy Propionibacterium extends the mean lifespan of *Caenorhabditis elegans* via activation of the innate immune system. Sci. Rep..

[42] Stiernagle T. (1999). Maintenance of *C. elegans*. C. Elegans.

[43] Yang J.-S., Nam H.-J., Seo M., Han S.K., Choi Y., Nam H.G., Lee S.-J., Kim S. (2011). OASIS: Online application for the survival analysis of lifespan assays performed in aging research. PLoS ONE.

[44] Soukas A.A., Kane E.A., Carr C.E., Melo J.A., Ruvkun G. (2009). Rictor/TORC2 regulates fat metabolism, feeding, growth, and life span in *Caenorhabditis elegans*. Genes Dev..

[45] Zanni E., Laudenzi C., Schifano E., Palleschi C., Perozzi G., Uccelletti D., Devirgiliis C. (2015). Impact of a complex food microbiota on energy metabolism in the model organism *Caenorhabditis elegans*. BioMed Res. Int..

[46] Chelliah R., Choi J.-G., Hwang S.-b., Park B.-J., Daliri E.B.-M., Kim S.-H., Wei S., Ramakrishnan S.R., Oh D.-H. (2018). In vitro and in vivo defensive effect of probiotic LAB against *Pseudomonas aeruginosa* using *Caenorhabditis elegans* model. Virulence.

[47] Bendesky A., Tsunozaki M., Rockman M.V., Kruglyak L., Bargmann C.I. (2011). Catecholamine receptor polymorphisms affect decision-making in *C. elegans*. Nature.

[48] Zhou L., Fu X., Luo Y., Du F., Wang H., Xing S., Li W., Ma J. (2017). 2-SeCD treatment extends lifespan, improves healthspan and enhances resistance to stress in *Caenorhabditis elegans*. RSC Adv..

[49] Lee H., Cho J.S., Lambacher N., Lee J., Lee S.-J., Lee T.H., Gartner A., Koo H.-S. (2008). The *Caenorhabditis elegans* AMP-activated protein kinase AAK-2 is phosphorylated by LKB1 and is required for resistance to oxidative stress and for normal motility and foraging behavior. J. Biol. Chem..

[50] Kim J., Moon Y. (2019). Worm-based alternate assessment of probiotic intervention against gut barrier infection. Nutrients.

[51] Livak K.J., Schmittgen T.D. (2001). Analysis of relative gene expression data using real-time quantitative PCR and the 2− ΔΔCT method. Methods.

[52] Yoon D.S., Lee M.-H., Cha D.S. (2018). Measurement of Intracellular ROS in *Caenorhabditis elegans* Using 2′, 7′-Dichlorodihydrofluorescein Diacetate. Bio-Protocol.

[53] Dilberger B., Baumanns S., Schmitt F., Schmiedl T., Hardt M., Wenzel U., Eckert G.P. (2019). Mitochondrial oxidative stress impairs energy metabolism and reduces stress resistance and longevity of *C. Elegans*. Oxidative Med. Cell. Longev..

[54] Ganguly N., Bhattacharya S., Sesikeran B., Nair G., Ramakrishna B., Sachdev H., Batish V., Kanagasabapathy A., Muthuswamy V., Kathuria S. (2011). ICMR-DBT guidelines for evaluation of probiotics in food. Indian J. Med. Res..

[55] Park M.R., Oh S., Son S.J., Park D.-J., Oh S., Kim S.H., Jeong D.-Y., Oh N.S., Lee Y., Song M. (2015). Bacillus licheniformis isolated from traditional Korean food resources enhances the longevity of *Caenorhabditis elegans* through serotonin signaling. J. Agric. Food Chem..

[56] Li Z., Zhang Z., Ren Y., Wang Y., Fang J., Yue H., Ma S., Guan F. (2021). Aging and age-related diseases: From mechanisms to therapeutic strategies. Biogerontology.

[57] World Health Organization (2015). World Report on Ageing and Health.

[58] Jena P.K., Trivedi D., Thakore K., Chaudhary H., Giri S.S., Seshadri S. (2013). Isolation and characterization of probiotic properties of lactobacilli isolated from rat fecal microbiota. Microbiol. Immunol..

[59] Roselli M., Schifano E., Guantario B., Zinno P., Uccelletti D., Devirgiliis C. (2019). *Caenorhabditis elegans* and probiotics interactions from a prolongevity perspective. Int. J. Mol. Sci..

[60] Zhou M., Liu X., Yu H., Gong J. (2021). Lactobacillus Regulates *Caenorhabditis elegans* Cell Signaling to Combat Salmonella Infection. Front. Immunol..

[61] Marchionni S., Sell C., Lorenzini A. (2020). Development and Longevity: Cellular and Molecular Determinants—A Mini-Review. Gerontology.

[62] Lee W.-S., Monaghan P., Metcalfe N.B. (2013). Experimental demonstration of the growth rate–lifespan trade-off. Proc. R. Soc. B Biol. Sci..

[63] Saul N., Möller S., Cirulli F., Berry A., Luyten W., Fuellen G. (2021). Health and longevity studies in *C. elegans*: The “healthy worm database” reveals strengths, weaknesses and gaps of test compound-based studies. Biogerontology.

[64] Johnson A.A., Stolzing A. (2019). The role of lipid metabolism in aging, lifespan regulation, and age-related disease. Aging Cell.

[65] Van Gilst M.R., Hadjivassiliou H., Jolly A., Yamamoto K.R. (2005). Nuclear hormone receptor NHR-49 controls fat consumption and fatty acid composition in *C. elegans*. PLoS Biol..

[66] Brock T.J., Browse J., Watts J.L. (2007). Fatty acid desaturation and the regulation of adiposity in *Caenorhabditis elegans*. Genetics.

[67] Han S., Schroeder E.A., Silva-García C.G., Hebestreit K., Mair W.B., Brunet A. (2017). Mono-unsaturated fatty acids link H3K4me3 modifiers to *C. elegans* lifespan. Nature.

[68] Tsui D., van der Kooy D. (2008). Serotonin mediates a learned increase in attraction to high concentrations of benzaldehyde in aged *C. elegans*. Learn. Mem..

[69] Rouse J., Cohen P., Trigon S., Morange M., Alonso-Llamazares A., Zamanillo D., Hunt T., Nebreda A.R. (1994). A novel kinase cascade triggered by stress and heat shock that stimulates MAPKAP kinase-2 and phosphorylation of the small heat shock proteins. Cell.

[70] Freshney N.W., Rawlinson L., Guesdon F., Jones E., Cowley S., Hsuan J., Saklatvala J. (1994). Interleukin-1 activates a novel protein kinase cascade that results in the phosphorylation of Hsp27. Cell.

[71] Han J., Lee J., Bibbs L., Ulevitch R. (1994). A MAP kinase targeted by endotoxin and hyperosmolarity in mammalian cells. Science.

[72] Kim D.H., Feinbaum R., Alloing G., Emerson F.E., Garsin D.A., Inoue H., Tanaka-Hino M., Hisamoto N., Matsumoto K., Tan M.-W. (2002). A conserved p38 MAP kinase pathway in *Caenorhabditis elegans* innate immunity. Science.

[73] Sifri C.D., Begun J., Ausubel F.M., Calderwood S.B. (2003). *Caenorhabditis elegans* as a model host for *Staphylococcus aureus* pathogenesis. Infect. Immun..

[74] Mallo G.V., Kurz C.L., Couillault C., Pujol N., Granjeaud S., Kohara Y., Ewbank J.J. (2002). Inducible antibacterial defense system in *C. elegans*. Curr. Biol..

[75] Roeder T., Stanisak M., Gelhaus C., Bruchhaus I., Grötzinger J., Leippe M. (2010). Caenopores are antimicrobial peptides in the nematode *Caenorhabditis elegans* instrumental in nutrition and immunity. Dev. Comp. Immunol..

[76] Miltsch S.M., Seeberger P.H., Lepenies B. (2014). The C-type lectin-like domain containing proteins Clec-39 and Clec-49 are crucial for *Caenorhabditis elegans* immunity against *Serratia marcescens* infection. Dev. Comp. Immunol..

[77] Kato Y., Aizawa T., Hoshino H., Kawano K., Nitta K., Zhang H. (2002). abf-1 and abf-2, ASABF-type antimicrobial peptide genes in *Caenorhabditis elegans*. Biochem. J..

[78] Chen W.-Y., Wang M., Zhang J., Barve S.S., McClain C.J., Joshi-Barve S. (2017). Acrolein disrupts tight junction proteins and causes endoplasmic reticulum stress-mediated epithelial cell death leading to intestinal barrier dysfunction and permeability. Am. J. Pathol..

[79] Hsieh C.Y., Osaka T., Moriyama E., Date Y., Kikuchi J., Tsuneda S. (2015). Strengthening of the intestinal epithelial tight junction by *Bifidobacterium bifidum*. Physiol. Rep..

[80] Liguori I., Russo G., Curcio F., Bulli G., Aran L., Della-Morte D., Gargiulo G., Testa G., Cacciatore F., Bonaduce D. (2018). Oxidative stress, aging, and diseases. Clin. Interv. Aging.

[81] Weydert C.J., Cullen J.J. (2010). Measurement of superoxide dismutase, catalase and glutathione peroxidase in cultured cells and tissue. Nat. Protoc..

[82] Lithgow G.J., Walker G.A. (2002). Stress resistance as a determinate of *C. elegans* lifespan. Mech. Ageing Dev..

[83] Tower J. (2011). Heat shock proteins and Drosophila aging. Exp. Gerontol..

[84] Barja G. (2014). The mitochondrial free radical theory of aging. Progress in Molecular Biology and Translational Science.

[85] Bhatti J.S., Bhatti G.K., Reddy P.H. (2017). Mitochondrial dysfunction and oxidative stress in metabolic disorders—A step towards mitochondria based therapeutic strategies. Biochim. Biophys. Acta-Mol. Basis Dis..

[86] Wang C., Youle R.J. (2009). The role of mitochondria in apoptosis. Annu. Rev. Genet..

