# Supplementary material for: A Potential Probiotic Lactobacillus plantarum JBC5 Improves Longevity and Healthy Aging by Modulating Antioxidative, Innate Immunity and Serotonin-Signaling Pathways in Caenorhabditis elegans
Source: Antioxidants (Basel). 2022 Jan 28;11(2):268. doi: 10.3390/antiox11020268 (PMC8868178; doi:10.3390/antiox11020268)
Supplement: Supplementary file 1 [file antioxidants-11-00268-s001.zip › antioxidants-1499811-supplementary.pdf]

A potential probiotic *Lactobacillus plantarum* JBC5 improves longevity and healthy aging by modulating antioxidative, innate immunity and serotonin-signaling pathways in *Caenorhabditis elegans*

Arun Kumar[1], Tulsi Joishy[1], Santanu Das[1], Mohan C. Kalita[2], Ashis K. Mukherjee[1,3,] and Mojibur R. Khan[1]*

[1]Molecular Biology and Microbial Biotechnology Laboratory, Division of Life Sciences, Institute of Advanced Study in Science and Technology (IASST), Guwahati-781035, Assam, India.

[2]Department of Biotechnology, Gauhati University, Guwahati-781014, Assam, India

[3]Department of Molecular Biology and Biotechnology, School of Sciences, Tezpur University, Tezpur-784028, Assam, India

*Corresponding author's email: mojibur.khan@iasst.gov.in

Supplementary material

Figure S1

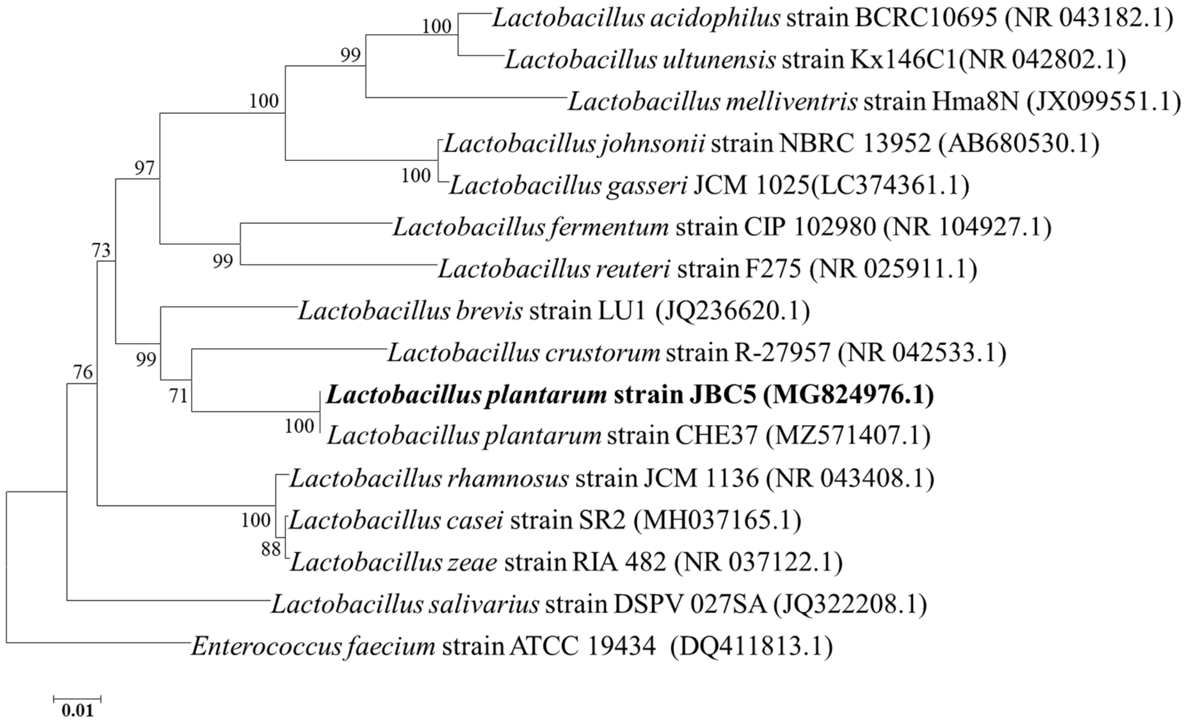

Figgure S1. Neighbour-joining tree was based on 16S rRNA gene sequences (1297 bases) of *L. plantarum* JBC5 (Accession no.: MG824976.1) and other related species of *Lactobacillus*. Bootstrap values (expressed as percentages of 1000 replications) greater than 60% are given at nodes. GenBank accession number are provided in the parentheses of each strain. *Enterococcus faecium* ATCC 19434 (Accession no.: DQ411813.1) was used as an outgroup. The evolutionary distances were computed using the Kimura 2-parameter method in MEGA 7 software, representing the number of base substitutions per site. The bar represents 0.01 substitutions per nucleotide position.

Figure S2

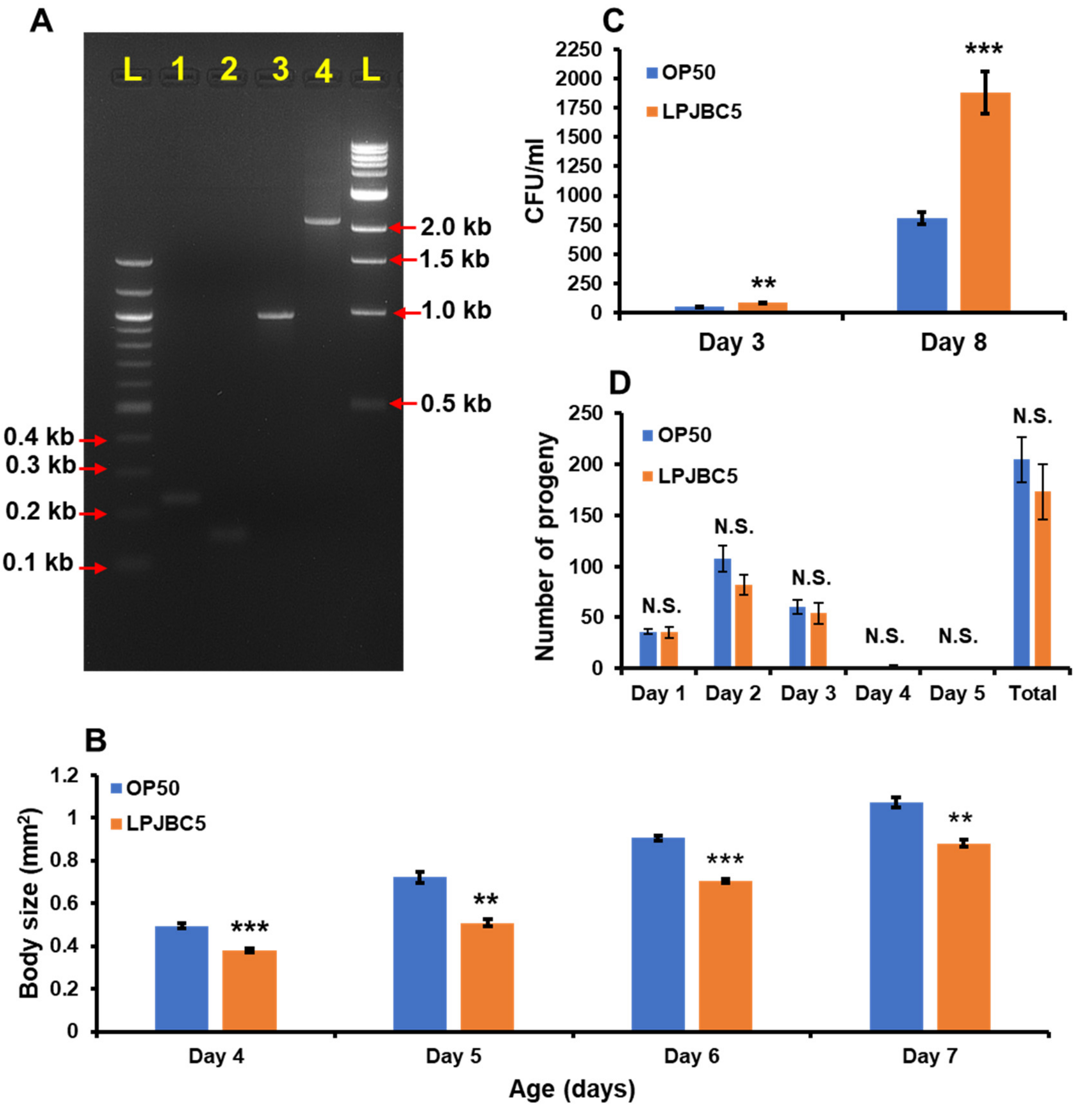

Figure S2 **A** An agarose gel image of PCR amplified products of *Lactobacillus plantarum* specific antimicrobial plantaricin-biosynthetic gene (*Pln*) PlnF/R (lane 1: ~231 bp) (Acc. no.: MW846638), species-specific segment PlantarumF/R (lane 2: ~152 bp) (Acc. no.: MW846639), probiotic marker genes (i.e., bile salt hydrolase (*Lpbsh1*) (lane 3: ~975 bp) (Acc. no.: MW846636) and collagen-binding protein (*Lpcbp*) (lane 4: ~2174) (Acc. no.: MW846637) **B.** Effect of LPJBC5 on the body size of worms (***$p < 0.0001$, log-rank test). **C and D.** The colonization efficiency and brood size were analyzed after feeding OP50 or LPJBC5. Error

bars represent mean ± SEM. Treatment effects were compared using Student's t-test (**p < 0.01 and ***p < 0.001).

Figure S3

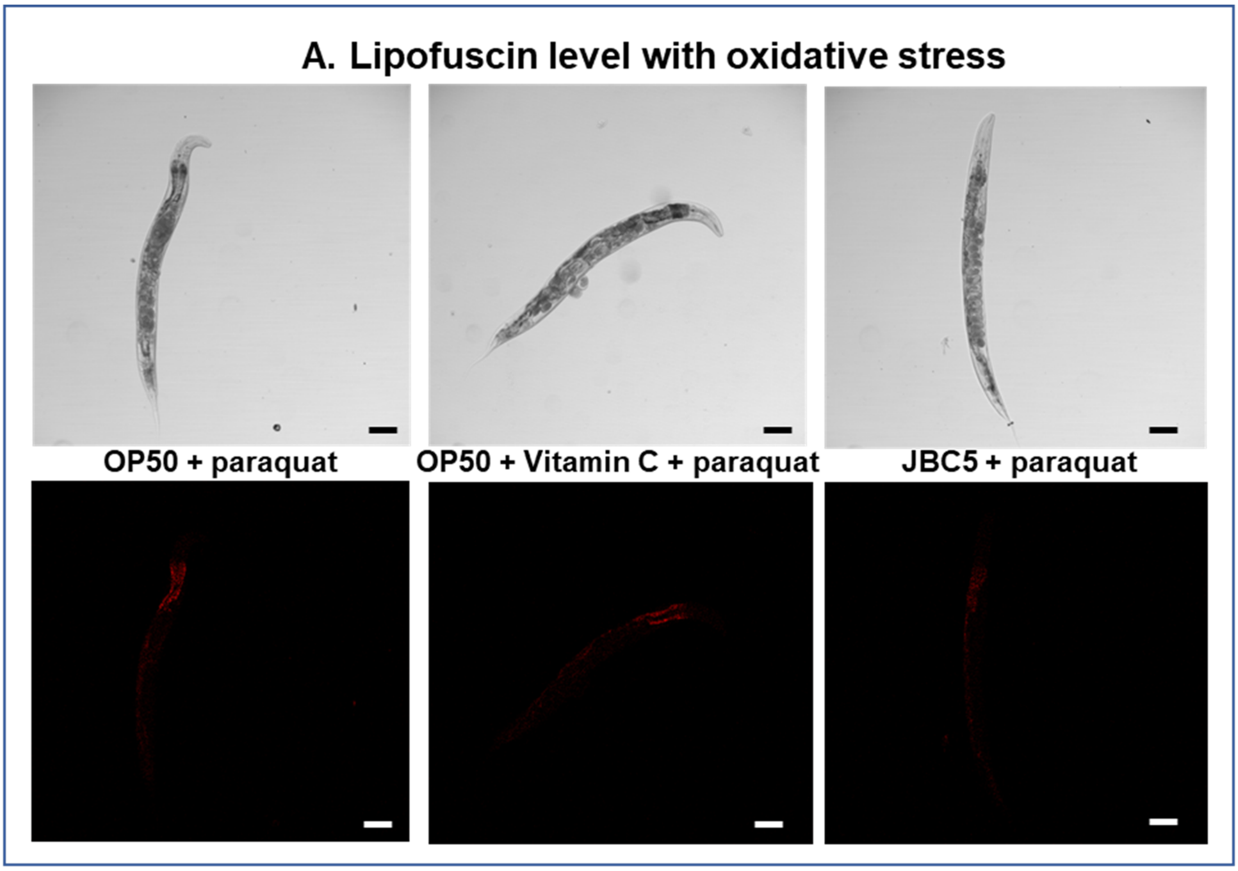
A. Lipofuscin level with oxidative stress
OP50 + paraquat
OP50 + Vitamin C + paraquat
JBC5 + paraquat

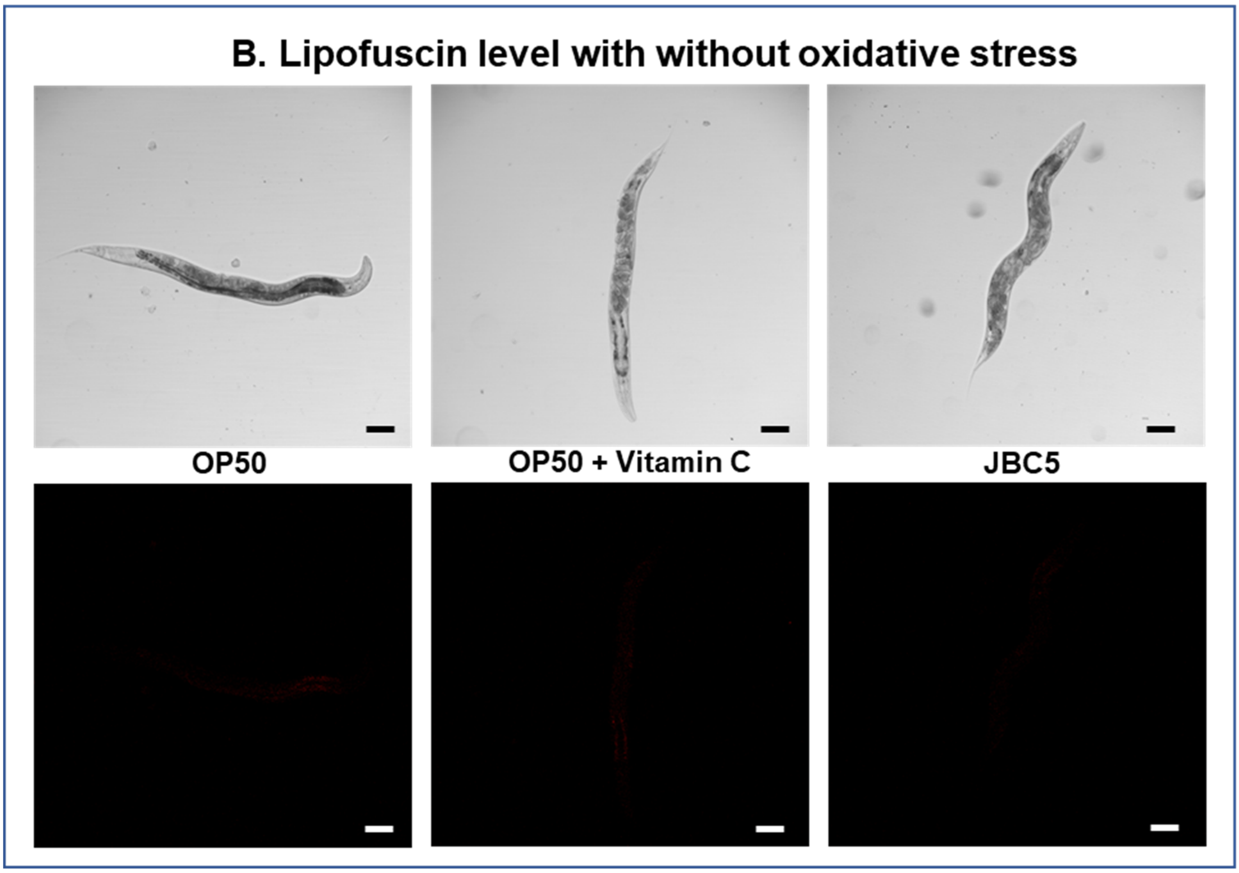
B. Lipofuscin level with without oxidative stress
OP50
OP50 + Vitamin C
JBC5

Figure S3. Accumulation of lipofuscin (i.e. a measure of senescence) with and without exposure to oxidative stress (100 mM paraquat) was observed under a confocal microscope at 10X magnification (Scale bar, 100 μm).

Figure S4

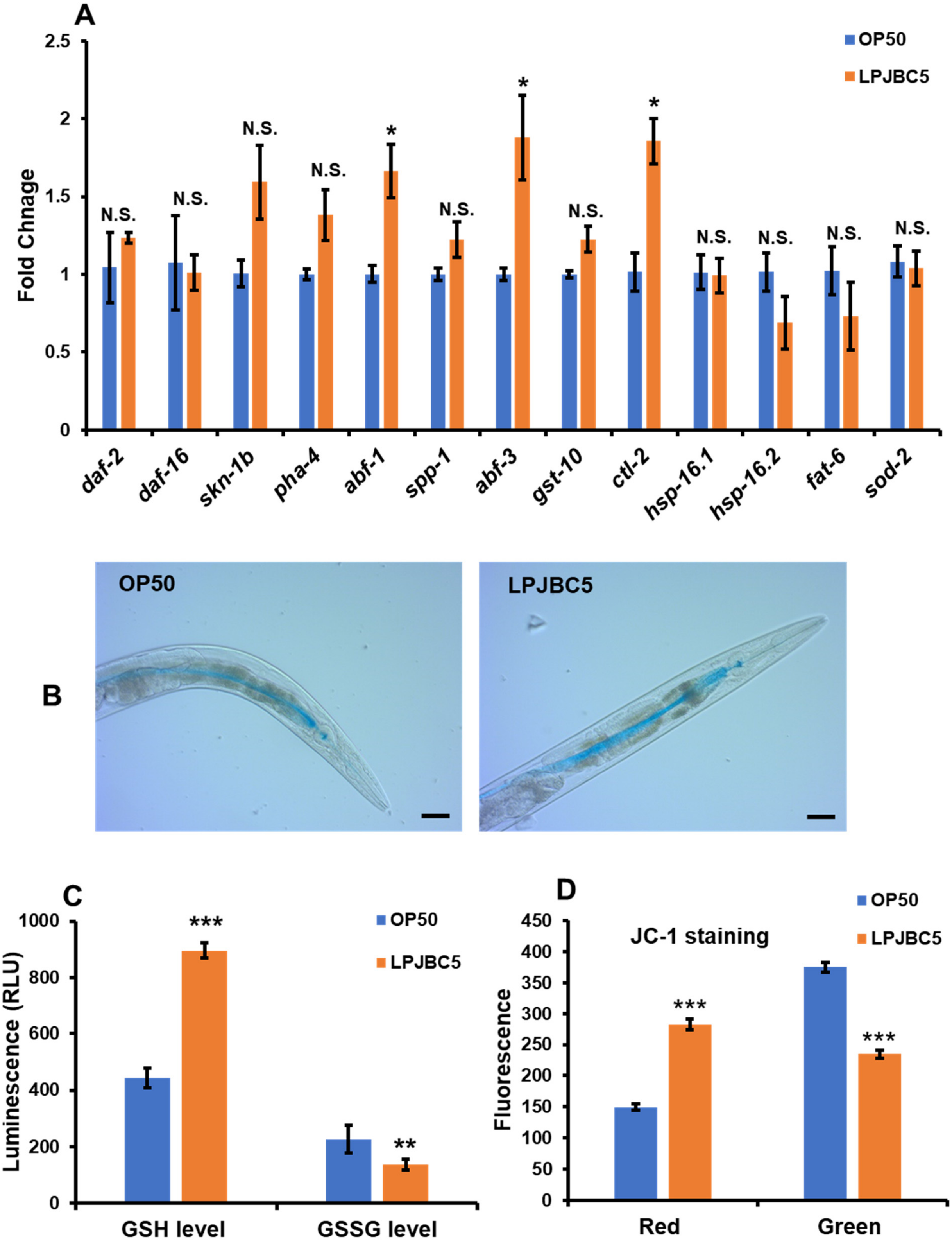

A
OP50
LPJBC5
Fold Chnage
N.S.
*
daf-2
daf-16
skn-1b
pha-4
abf-1
spp-1
abf-3
gst-10
ctl-2
hsp-16.1
hsp-16.2
fat-6
sod-2
B
OP50
LPJBC5
C
Luminescence (RLU)
***
**
GSH level
GSSG level
D
JC-1 staining
Fluorescence
Red
Green

Figure S4 A. qRT-PCR analysis on the expression of genes involved in longevity, stress resistance, immunity, and fat accumulation. **B.** The intestinal integrity of worms was observed in control groups OP50 and LPJBC5 under a compound microscope at 20X (Scale bar, 20 μm). The feeding of LPJBC5 improves GSH, reduces GSSG level **(C)**, improves mitochondrial membrane potential in worms **(D).** Error bars represent mean ± SEM. Treatment effects were compared using Student's t-test (*$p < 0.05$, **$p < 0.01$ and ***$p < 0.001$).

Table S1 Primers used to characterize the LPJBC5

***L. plantarum* specific gene**	**Accession number**	**Primer**	**Sequence**	Reference
***Plantarum* (Species-specific sequence)**	MW846639	forward	5'- GCTGGCAATGCCATCGTGCT -3'	[1]
		reverse	5'- TCTCAACGGTTGCTGTATCG -3'	
Lpbsh1 (Bile salt hydrolase gene)	MW846636	forward	5'- ATGTGTACTGCCATAACTT -3'	[2]
		reverse	5'- TTAGTTAACTGCATAGTATTG -3'	
Lpcbp (Collagen binding protein)	MW846637	forward	5'- ATGGGGGAGGAGCGTATG -3'	[2]
		reverse	5'- AGCACGACGGCGATAA -3'	
***Pln* (Plantaricin protein)**	MW846638	forward	5'- TTGCCACCAACCATTCAGC -3'	[3]
		reverse	5'- CTAACCATGATGATAATCGA -3'	

References

1. Kim, E., et al., Design of PCR assays to specifically detect and identify 37 *Lactobacillus* species in a single 96 well plate. BMC microbiology, 2020. **20**: p. 1-14.
2. Kumar, R., S. Grover, and V.K. Batish, Molecular identification and typing of putative probiotic indigenous *Lactobacillus plantarum* strain Lp91 of human origin by specific primed-PCR assays. Probiotics and antimicrobial proteins, 2011. **3**(3-4): p. 186-193.
3. Khemariya, P., et al., Isolation and identification of *Lactobacillus plantarum* from vegetable samples. Food biotechnology, 2016. **30**(1): p. 49-62.

Table S2 Primer sets used for qRT-PCR analysis.

Gene Name (Accession no.)	Primer	Sequence	References
sek-1	forward	5'-GCCGATGGAAAGTGGTTTTA-3'	[1]
(AB024087)	reverse	5'-TAAACGGCATCGCCAATAAT-3'	
nsy-1	forward	5'-AGTTGTAGACACGCCGGTTA-3'	[1]
(NM_001383826)	reverse	5'-TGCACTGTTCAGGCTTTCAC-3'	
pmk-1	forward	5'-CCGACTCCACGAGAAGGATA-3'	[1]
(NM_068964)	reverse	5'-AGCGAGTACATTCAGCAGCA-3'	
skn-1	forward	5'-CTCTCTTCTGGCATCCTCTACCA-3'	[1]
(NM_171347)	reverse	5'-TTCTTGGATTCTTCTTCTTGTTCGT-3'	
skn-1b	forward	5'-GGCAGCAACCTTGTTCTTTC-3'	[1]
(NM_001380238)	reverse	5'-GGACGTCAACAGCAGACTCA-3'	
daf-2	forward	5'-AAAAGATTTGGCTGGTCAGAGA-3'	[1]
(NM_001312989)	reverse	5'-TTTCAGTACAAATGAGATTGTCAGC-3'	
daf-16	forward	5'-TTCAATGCAAGGAGCATTTG-3'	[1]
(NM_001377628)	reverse	5'-AGCTGGAGAAACACGAGACG-3'	
**act-1**	forward	5'-GCTGGACGTGATCTTACTGATTACC-3'	[1]
(NM_073418)	reverse	5'-GTAGCAGAGCTTCTCCTTGATGTC-3'	
sod-1	forward	5'-CGTAGGCGATCTAGGAAATGTG-3'	[1]
(NM_001026785)	reverse	5'-AACAACCATAGATCGGCCAACG-3'	
sod-2	forward	5'-CTTCAAAACACCGTTCGCTG-3'	[1]
(NM_059889)	reverse	5'-CAGTGGAACAAGTCCAGTT-3'	
sod-3	forward	5'-TTCAAAGGAGCTGATGGACACT-3'	[1]
(NM_078363)	reverse	5'-AAGTGGGACCATTCCTTCCAA-3'	
trx-1	forward	5'-TCCAACACTTTTTGACGCAG-3'	[1]

(NM_001026714)	reverse	5'-CAAGATGATGCCGACTTTCA-3'	
tph-1	forward	5'-AAGAGGCCCAGCAGAAACTC-3'	[2]
(NM_001373740)	reverse	5'-ATGGAACGGGAGTTGTTGAG-3'	
ser-1	forward	5'-AAGAGCCAGTCGCCAGAAC-3'	[2]
(NM_001029558)	reverse	5'-GTGGTTGATGCCTCTGTCGT-3'	
mod-1	forward	5'-GAAGCAACGGGTATGCAA-3'	[2]
(AF303088)	reverse	5'-CCGTTTCGATGAAGTGATCC-3'	
fat-5	forward	5'-TGGTGAAGAAGCACGATCAG-3'	[3]
(NM_075081)	reverse	5'-AAGCAGAAGATTCCGACCAA-3'	
fat-6	forward	5'- CTTGTGCTGCTTCATTCTTCC-3'	[4]
(NM_001268668)	reverse	5'- GAAGTTGTGACCTCCCTCTCC-3'	
fat-7	forward	5'- ACCCGTGGATTCTTCTTCACT-3'	[4]
(NM_072413)	reverse	5'- TAACGGAATGTTCCAGCTACG-3'	
hsp-16.1	forward	5'- GCAGAGGCTCTCCATCTGAA-3'	[3]
(NM_072956)	reverse	5'- GCTTGAACTGCGAGACATTG-3'	
hsp-16.2	forward	5'- CTATTTCCGTCCAGCTCAAC -3'	[1]
(NM_001392482)	reverse	5'- TTTGTTCAACGGGCGCTTGC -3'	
hsp-60	forward	5'- CTATGGGCCCAAAAGGAAGAAACGTG -3'	[5]
(NM_065028)	reverse	5'- GGATTTCGCGACGGTGACTCCGTCC -3'	
hsp-70	forward	5'-GAAAGGTTGAAATCCTCGCGAACTC -3'	[1]
(NM_060084)	reverse	5'- TCCGGATTACGAGCGGCTTGATCTT -3'	
Ctl-1	forward	5'- CGGATACCGTACTCGTGATGA -3'	[1]
(U55384)	reverse	5'- CCAAACAGCCACCCAAATCA -3'	
Ctl-2	forward	5'- TCCGTGACCCTATCCACTTC -3'	[1]
(NM_001027302)	reverse	5'- TGGGATCCGTATCCATTCAT -3'	
gst-4	forward	5'- GATGCTCGTGCTCTTGCTG -3'	[1]

(NM_069447)	reverse	5'- CCGAATTGTTCTCCATCGAC -3'	
gst-7	forward	5'- GGACAAGACTTCGAGGACAAC -3'	[1]
(NM_062482)	reverse	5'- AACTGACGAGCCAAGTAACG -3'	
gst-10	forward	5'- AAGAGATTGTGCAGACTGGAG -3'	[1]
(NM_071300)	reverse	5'- AGAACATGTCGAGGAAGGTTG -3'	
abf-1	forward	5'- GTACAGCACAGAAATGCATGACCGG -3'	[6]
(AB029809)	reverse	5'- GGCGTTTGAACAACCTCCACAGAAGC -3'	
abf-2	forward	5'- CCGTTCCCTTTTCCTTGCAC -3'	[7]
(AB029810)	reverse	5'- GACGACCGCTTCGTTTCTTG -3'	
abf-3	forward	5'- AACAGATTGGGGTCAGCTCG -3'	[7]
(NM_074549)	reverse	5'- TGGAGACCATTATTGCCGGG -3'	
spp-1	forward	5'- GGCTCTCGTCGAGGGTGGAGAG -3'	[7]
(NM_066889)	reverse	5'- CACACTCGTGATGCAACGGCAACAGC -3'	
spp-7	forward	5'-CAGTACGAACTACAACAGCTTTGACTCG-3'	[6]
(NM_068407)	reverse	5'- GTATTTTGAACGAAGCGGTGGTGG -3'	
lys-1	forward	5'- GGATTCAGGTTACCTCCCCAGCC -3'	[6]
(NM_073241)	reverse	5'- GGTGTAGATTCCGACAGTCAGTCCG -3'	
lys-7	forward	5'- GCGGGTTATTGTGCAGTTTT -3'	[3]
(NM_071571)	reverse	5'- TCAATTCCGAGTCCAGCTTT -3'	
clec-60	forward	5'- CGGTTTCAATGCGGTATGGC -3'	[7]
(NM_063858)	reverse	5'- TGAAGCTGTGGTTGAGGCAT -3'	
clec-85	forward	5'- CCAATGGGATGACGGAACCA -3'	[7]
(NM_001383060)	reverse	5’- CTTCTGTCCAGCCAACGTCT -3’	
ced-3	forward	5’- ACGGGAGATCGTGAAAGC -3’	[8]
(AF210702)	reverse	5'- AGAGTTGGCGGATGAAGG -3'	

ced-4	forward	5'- AGTCACTCGCAATGGCTCT -3'	[8]
(NM_001026032)	reverse	5'- GCTGATGAACGACGGAAT -3'	
ced-9	forward	5'- AAAGGCACAGAGCCCACC -3'	[8]
(NM_066883)	reverse	5'- CGTTCCCATAACTCGCATC -3'	
nd-1	forward	5'- AGCGTCATTTATTGGGAAGAAGAC -3'	[9]
(AY171147)	reverse	5'- AAGCTTGTGCTAATCCCATAAATGT -3'	
zoo-1	forward	5'- ATTCGGTGGGACAGTTGGTC -3'	[10]
(NM_001265448)	reverse	5'- CGGGTCTATGGAACGATGGG -3'	

References

1. Nakagawa, H., et al., Effects and mechanisms of prolongevity induced by *Lactobacillus gasseri* SBT2055 in *Caenorhabditis elegans*. Aging cell, 2016. **15**(2): p. 227-236.
2. Park, M.R., et al., *Bacillus licheniformis* isolated from traditional Korean food resources enhances the longevity of *Caenorhabditis elegans* through serotonin signaling. Journal of agricultural and food chemistry, 2015. **63**(47): p. 10227-10233.
3. Li, J., et al., *Caenorhabditis elegans* HCF-1 functions in longevity maintenance as a DAF-16 regulator. PLoS Biol, 2008. **6**(9): p. e233.
4. Lin, C., et al., Anti-fat effect and mechanism of polysaccharide-enriched extract from Cyclocarya paliurus (Batal.) Iljinskaja in *Caenorhabditis elegans*. Food & Function, 2020. **11**(6): p. 5320-5332.
5. Jeong, D.E., et al., Mitochondrial chaperone HSP-60 regulates anti-bacterial immunity via p38 MAP kinase signaling. The EMBO journal, 2017. **36**(8): p. 1046-1065.
6. Alper, S., et al., Specificity and complexity of the *Caenorhabditis elegans* innate immune response. Molecular and cellular biology, 2007. **27**(15): p. 5544-5553.

7. Zhou, M., et al., Cell signaling of *Caenorhabditis* elegans in response to enterotoxigenic *Escherichia coli* infection and *Lactobacillus zeae* protection. Frontiers in immunology, 2018. **9**: p. 1745.

8. Schertel, C. and B. Conradt, *C. elegans* orthologs of components of the RB tumor suppressor complex have distinct pro-apoptotic functions. 2007.

9. Kassahun, H., et al., Constitutive MAP-kinase activation suppresses germline apoptosis in NTH-1 DNA glycosylase deficient *C. elegans*. DNA repair, 2018. **61**: p. 46-55.

10. Kim, J. and Y. Moon, Worm-based alternate assessment of probiotic intervention against gut barrier infection. Nutrients, 2019. **11**(9): p. 2146.

Table S3 Survival and adhesion of LPJBC5 under *in vitro* simulated gastro-intestinal conditions.

Gastrointestinal juice tolerance											
**Treatment**	pH 1		pH 3		pH 7		Pepsin (pH 2)			Pancreatin (pH 8)	
**Time**	0 h	1 h	0 h	1 h	0 h	1 h	0 h	1 h	3 h	0 h	4 h
**Mean* ± SEM**	9.04 ± 0.08	8.53 ± 0.02	8.30 ± 0.010	8.24 ± 0.04	9.10 ± 0.06	9.06 ± 0.09	9.19 ± 0.01	8.58 ± 0.21	8.32 ± 0.17	9.18 ± 0.08	9.14 ± 0.03
**#Survival %**	94.35		99.27		99.56		93.36			99.56	
**P value**	$p < 0.05$		$p > 0.05$		$p > 0.05$		$p < 0.05$ for 1 h and 3 h			$p > 0.05$	

	Bile salt tolerance				Adhesion in HT-29 cell line	
**Treatment**	0.3%		1%			
**Time**	0 h	4 h	0 h	4 h	0 h	4 h
**Mean* ± SEM**	9.28 ± 0.16	9.10 ± 0.09	9.19 ± 0.03	8.95 ± 0.04	9.49 ± 0.18	8.79 ± 0.13
**#Survival %**	96.98		97.38		92.62	
**P value**	$p > 0.05$		$p < 0.05$		$p < 0.05$	

* The values represent mean log (CFU/ml) ± standard error mean (SEM).

# Survival percentage (%) is expressed as the percentage of 1– [(log CFU per ml at T= initial) – (log CFU per ml at T = incubation hour) / (log CFU per ml at T = initial)].

Table S4 Closest gene homologs of LPJBC5 in the NCBI database.

**Accession number**	**Genes of *L. plantarum***	**Closest homolog (% identity)**
**MW846636**	Bile salt hydrolase (*Lpbsh1*)	*Lactiplantibacillus plantarum* strain CNEI-KCA4 chromosome (CP053571.1) (100%)
**MW846637**	Collagen-binding protein (*Lpcbp*)	*Lactobacillus plantarum* strain 91 collagen binding gene (GQ340730.1) (100%)
**MW846638**	Plantaricin biosynthesis (*Pln*)	*Lactiplantibacillus plantarum* strain CNEI-KCA4 chromosome (CP053571.1) (100%)
**MW846639**	Species-specific sequence (*Plantarum*)	*Lactiplantibacillus plantarum* strain 83-18 chromosome (CP046669.1) (90.09%)
